# Efficient prospective electric field-informed localization of motor cortical targets of transcranial magnetic stimulation

**DOI:** 10.1162/IMAG.a.1056

**Published:** 2025-12-19

**Authors:** David Luis Schultheiss, Zsolt Turi, Srilekha Marmavula, Peter Christoph Reinacher, Theo Demerath, Jakob Straehle, Joschka Boedecker, Matthias Mittner, Andreas Vlachos

**Affiliations:** Neurobotics Lab, Department of Computer Science, University of Freiburg, Freiburg, Germany; Center BrainLinks-BrainTools, University of Freiburg, Freiburg, Germany; Department of Neuroanatomy, Institute of Anatomy and Cell Biology, Faculty of Medicine, University of Freiburg, Freiburg, Germany; Department of Stereotactic and Functional Neurosurgery, Medical Center - University of Freiburg, Faculty of Medicine, University of Freiburg, Freiburg, Germany; Fraunhofer Institute for Laser Technology (ILT), Aachen, Germany; Department of Neuroradiology, Medical Center-University of Freiburg, Faculty of Medicine, University of Freiburg, Freiburg, Germany; Department of Neurosurgery, Medical Center-University of Freiburg, Faculty of Medicine, University of Freiburg, Freiburg, Germany; Institute for Psychology, Norwegian University of Science and Technology, Trondheim, Norway; Institute for Psychology, UiT-The Arctic University of Norway, Tromsø, Norway; Center for Basics in NeuroModulation (NeuroModulBasics), Faculty of Medicine, University of Freiburg, Freiburg, Germany

**Keywords:** transcranial magnetic stimulation, electric field, motor mapping, motor evoked potentials, motor cortex, farthest point sampling

## Abstract

Transcranial magnetic stimulation (TMS) is a versatile non-invasive tool for brain mapping and neuromodulation in both healthy individuals and patients. Effective TMS-based causal brain mapping relies on precise localization of cortical targets. Current state-of-the-art approaches use statistical methods to quantify the relationship between TMS-induced electric fields (E-fields) and motor evoked potential (MEP) amplitudes. However, this method typically relies on the random selection of coil configurations, which limits its efficacy. In this study, we present a novel optimization strategy for TMS-based motor mapping by prospectively selecting coil configurations based on their E-field characteristics using an iterative sampling algorithm called farthest point sampling (FPS). Through a combination of theoretical analysis, simulation and experimental validation, including 10 healthy individuals, we systematically evaluated the performance of FPS against the random sampling approach. Our results demonstrate that FPS (median: 49.5 trials) is twice as sample-efficient as random sampling (median: 91.0 trials) in reducing the number of trials required to estimate the motor map. Moreover, FPS shows greater consistency across participants, as reflected in the narrower confidence intervals. These findings highlight the potential of FPS to significantly enhance the sample-efficiency of motor mapping, paving the way for the development of more effective TMS mapping algorithms.

## 1 Introduction

Transcranial magnetic stimulation (TMS) is a widely used interventional technique for the non-invasive stimulation of neural tissue in humans and animals (Allen et al., 2007; Barker et al., 1985; Mueller et al., 2014; Romero et al., 2019). TMS generates short-lasting intracranial electric fields (E-field) of approximately 200–300 μs duration (for bipolar pulses), reaching a strength of up to 150–200 V m^−1^ in the human neocortex (Barker et al., 1985; Laakso & Hirata, 2012; Thielscher et al., 2015; Turi et al., 2022; Wang et al., 2023). This E-field can depolarize neurons and, under certain conditions, trigger action potentials in specific subset of neurons (Siebner et al., 2022). TMS has emerged as a valuable tool for brain mapping in both neuroscience research and clinical applications, such as pre-surgical planning (Haddad et al., 2021; Krings et al., 1997, 2001; Picht et al., 2011; Raffa et al., 2019; Sollmann et al., 2016; Wassermann et al., 1992).

Brain mapping aims to establish causal links between brain regions and their associated functions. However, this task is complicated by the hierarchical organization of the brain, where brain regions are organized within larger functional networks (Batail et al., 2023; Castrillon et al., 2020; Opitz et al., 2016). Effective brain mapping requires the precise localization of cortical target–specific areas where TMS elicits measurable neuronal effects causally linked to an induced biological response. For instance, TMS applied to the human primary motor cortex can evoke muscle twitches in contralateral peripheral muscles, measurable as motor evoked potentials (MEPs) (Barker et al., 1985; Rothwell et al., 1987; Wassermann et al., 1992). Despite the widespread use of TMS-induced MEPs, the precise regions in the motor cortex responsible for these effects remain a topic of ongoing investigation (Bijsterbosch et al., 2012; Bungert et al., 2017; Fox et al., 2004; Krieg et al., 2015; Laakso et al., 2018; Reijonen et al., 2022; Siebner et al., 2022; Weise et al., 2020), limiting the effectiveness of TMS as a brain mapping tool. An improved understanding of how TMS impacts cortical targets can potentially enhance both neuroscience research and its application in clinical situations.

Various methods have been developed for TMS-based functional mapping of the motor cortex. These methods often focus on delineating motor cortical representations of peripheral muscles (hereafter referred to as muscle representations), such as the first dorsal interosseus muscle (FDI) (Wassermann et al., 1992; Weise et al., 2023). The conventional grid method involves stimulating a predefined grid of scalp sites, followed by calculating the center of gravity based on the evoked motor responses (Classen et al., 1998; Nazarova et al., 2021; Raffin et al., 2015; Wassermann et al., 1992). However, this method estimates muscle representations using locations correspondig to the scalp and cannot accurately capture their spatial dispersion within the motor cortex with the desired level of precision cf. (Weise et al., 2020).

State-of-the-art approaches aim to improve localization by statistically quantifying the relationship between the TMS-induced E-field and MEP amplitudes (Numssen et al., 2021; Weise et al., 2020, 2023). These methods typically involve the collection of MEPs generated by hundreds of stimulations at randomly selected coil positions, followed by retrospective E-field simulations at each location (Numssen et al., 2021; Weise et al., 2020, 2023). These approaches feature significant improvements both in terms of efficiency and spatial resolution compared to grid-based methods. However, since the random selection of stimulation site and coil location does not prioritize the most informative coil configuration, it can potentially take a large number of trials before the method converges on a solution with satisfactory precision. In particular, randomly selected coil positions frequently generate redundant E-field characteristics, unnecessarily increasing the number of trials needed for the mapping. Therefore, identifying optimal coil configurations needed for efficient and precise motor mapping is critical, particularly in clinical settings, where minimizing the experimental burden on patients is essential.

This study presents a novel, prospective E-field-informed TMS-based motor cortical mapping method, designed to improve efficiency. By employing the sampling algorithm *farthest point sampling* (FPS), we iteratively select coil configurations that are maximally dissimilar in terms of their absolute E-field magnitude (| E | ) distributions, promoting diverse spatial sampling within the region of interest. Our theoretical and experimental analyses congruently demonstrate that FPS significantly reduces the number of trials required for motor mapping compared to random sampling, offering a more sample-efficient approach beneficial for both research and clinical applications.

## 2 Methods

### 2.1 Study overview

In this study, we systematically evaluate the performance of the random sampling and the FPS algorithm through both, theoretical and experimental work. In the theoretical section, we develop a synthetic MEP generation model for in-silico simulations, allowing us to evaluate the performance of the two algorithms across various scenarios. In the subsequent experimental section—which constitutes the primary focus of this paper—we evaluate the performance of both algorithms in healthy individuals using real-time, neuronavigated, robotic-arm-guided TMS.

### 2.2 FPS algorithm

The goal of the experiment is to prospectively select coil configurations informed by their induced | E | within the region of interest (ROI). For a given coil configuration c, the simulated ROI | E | map is represented as ${\text{E}}_{\text{c}}$. The configuration for each pulse is a unique combination of coil location $x_{c},y_{c}$ and coil orientation $\alpha_{c}$. denoted as $\theta_{c}=\{x_{c},y_{c},\alpha_{c}\}$ Since the motor mapping experiment is limited to approximately a few hundred TMS trials per experimental session, a sampling algorithm is required to select a small subset $\text{S}=\{{\text{E}}_{1},...,{\text{E}}_{N_{s}}\}$ from a large candidate dataset $\text{C}={\text{E}}_{1},...,{\text{E}}_{N_{c}}\}$ . In theory, the size of the candidate set is infinite as coil location and orientation span a continuous parameter space. However, similar configurations produce similar | E | maps and we hypothesize that in order to decorrelate candidate locations in the ROI, dissimilar | E | maps are required. Thus, the parameter space is discretized, which allows sampling from a finite set of $N_{c}$ | E | map candidates. Depending on the resolution of the potential coil center distances and orientation angles, this could result in millions of coil configurations and, consequently, millions of potential | E | distributions. However, as shown in Supplementary Appendix A, coarser resolutions suffice, reducing $N_{c}$ to 1017.

**Algorithm 1:** Farthest Point Sampling

**input:** $\text{C}=\{{\text{E}}_{1},...,{\text{E}}_{N_{c}}\},\;N_{s}$ **output:** $\text{S}=\{{\text{E}}_{1},...,{\text{E}}_{N_{s}}\}$ **1** Randomly choose first sample $E_{1}\in \text{C}$ and add to S; **2 n=1 **; **3 while** $n<N_{s}$ **do** **4** select new sample $s_{n}={\text{argmax}}_{{\text{E}}_{c}\in \text{C}\text{\textbackslash{}}\text{S}}\;\underset{{\text{E}}_{j}\in \text{S}}{\text{min}}\left\|\;{\text{E}}_{c}-{\text{E}}_{j}\right\|$ ; **5** add ${\text{E}}_{{\text{s}}_{n}}$ to *S*; **6 n=n+1 **; **7 end**

A well-known and data-efficient iterative sampling technique is FPS (Eldar et al., 1997; Mitchell, 1991). The idea of FPS is to select a small subset from a larger dataset while ensuring that the selected samples are representative of the original set. In the present case, we choose sequential coil configurations that generate maximally dissimilar | E | in the ROI.

The first sample is chosen randomly from C. Then, the algorithm iteratively adds | E | maps to the subset by choosing the | E | map that is maximally dissimilar from the ones already selected. Hereby, ${\text{E}}_{s_{n}}$ is considered to be maximally dissimilar at step n, if its minimal Euclidean distance to previously sampled | E | maps is maximized across all remaining candidate | E | maps. This can be formulated as the following maximization condition for selecting $s_{n}$:

$$s_{n}=\underset{{\text{E}}_{c}\in \text{C}\text{\textbackslash{}}\;\text{S}}{\text{argmax}}\;\;\;\underset{{\text{E}}_{j}\in \text{S}}{\text{min}}||{\text{E}}_{c}-{\text{E}}_{j}||.$$ (1)

Here, ‖ . ‖ denotes the Euclidean norm. A pseudocode of FPS is presented in Algorithm 1.

### 2.3 Synthetic MEP simulations

**Fig. 1.**
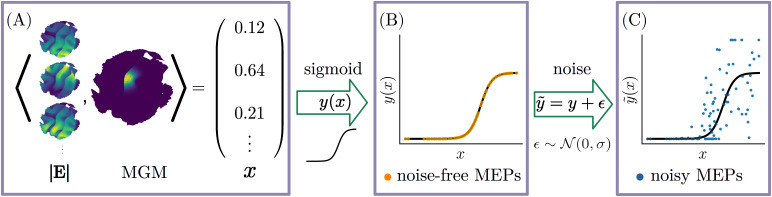
Flow chart of synthetic motor evoked potential (MEP) amplitude generation. (A) The stimulation strength x is computed as the inner product between a user defined MEP generating map (MGM) and E-field magnitude (| E | ) maps, one per coil configuration. Therefore, x does not directly correspond to the stimulation strength applied by any TMS device. Warmer colors of | E | and MGM indicate relatively higher values, and cooler colors indicate relatively lower values. The color mappings are illustrative, with all values shown on a relative scale. (B) The resulting x is passed through a sigmoid activation function to produce noise-free MEP amplitudes y. (C) Gaussian noise ϵ is added to generate noisy MEP amplitudes $\tilde{y}$ reflecting variability typically observed in experimental data. All MEP amplitudes $\tilde{y}$ are clipped to zero to ensure non-negativity.

We conducted a comprehensive simulation-based analysis based on synthetic MEPs to evaluate the performance of the two algorithms. Synthetic MEP simulations were based on a biologically inspired MEP generation map (MGM) and an MEP model. This simulation setup allows us to i) sample MEPs for arbitrary coil configurations, ii) place hypothetical muscle representations at different anatomical locations in motor cortex (e.g., gyral crown, gyral lip, sulcal wall, center of ROI, edge of ROI), and iii) study the effect of noise on the estimation of the motor map. The goal of the theoretical work was to investigate whether the sampling efficiency of the random method could be improved using the FPS approach. To this end, we dedicated substantial effort to ensuring that the synthetic model is sufficiently sensitive to detect meaningful differences between the two methods. While the synthetic framework incorporates several biologically inspired features—such as variability in MEP amplitudes, their distribution, and a plausible input–output function—it does not fully capture biological realism in other respects. For example, stimulation intensities do not directly correspond to actual device parameters, and MEP morphology is not modeled (see Fig. 1). Importantly, the theoretical work was conducted prior to the experimental study, and as such, the synthetic model was not validated against empirical data—nor was it intended to be. Rather, its purpose was to provide a controlled setting in which to explore and compare the relative performance of the sampling strategies.

#### 2.3.1 MEP generation map

We hypothesize the existence of a spatially restricted muscle representation in the motor cortex which is responsible for generating MEPs in that specific muscle. In our high-resolution representation of the human head, the muscle representation is discretized into K discrete compartments. These compartments are triangles if the ROI is defined on the brain surface or tetrahedrons if the ROI is a brain volume. For our simulations, we position the muscle representation at any compartment $k_{\text{max}}\in K$ , referred to as the most excitable compartment. Since the exact size and boundaries of the muscle representation in human motor cortex are uncertain, we model excitability as a distribution centered around the most excitable location $k_{\text{max}}$ . A Gaussian probability density function, which decays exponentially with distance from its center, is a suitable choice for this distribution. Based on this, the excitability $m_{k}$ of compartment k is defined as

$$m_{k}=\frac{1}{\sigma \sqrt{2\pi }}\text{exp}\left(-\frac{1}{2}\frac{d{\left(k,k_{\text{max}}\right)}^{2}}{\sigma^{2}}\right)$$ (2)

where $d(k,k_{\text{max}})$ is the geodesic distance on the cortex of compartment k from the most excitable location $k_{\text{max}}$ . The spatial extent of the excitable area is controlled by the standard deviation of the Gaussian distribution σ. The excitabilities of all compartments are summarized in the MGM M. This approach assumes that excitability decreases smoothly as the distance from the most excitable location increases, forming a focused excitability profile.

#### 2.3.2 MEP model

An underlying assumption guiding motor mapping within the primary motor cortex is the presence of a sigmoidal relationship between stimulation strength x in an excitable location of the cortex and MEP amplitude y (Numssen et al., 2021; Weise et al., 2023). The sigmoid function takes the form

$$y(x,\psi )=\frac{y_{max}}{1+e^{-k(x-x_{0})}},$$ (3)

where $x_{0}$ is the location of the turning point on the abscissa, $y_{max}$ is the saturation amplitude, and k is the slope. The parameters are summarized in $\psi =\{x_{0},y_{max},k\}$ .

We define the stimulation strength x as the inner product of the MGM M and the stimulus | E | map E:

x=〈M,E〉. (4)

This reflects the assumption, that strong | E | that overlap the muscle representation will increase the measured MEP while activations outside the MGM will not affect it. Then, zero-mean Gaussian noise ϵ is added to mimic variability stemming from a variety of sources (e.g., small coil or head movements, brain-state, etc.) inherent to experimental measurements, ensuring that the simulated data more accurately reflect real-world conditions:

$$\tilde{y}\left(x,\psi \right)=y\left(x,\psi \right)+\epsilon .$$ (5)

We observed in previously recorded data that noise tends to be stronger for a higher stimulation intensities. However, it also seems to saturate for very strong | E | . Therefore, the noise is modulated by a sigmoid term and noise amplitude P and ϵ→ϵ(x,ψ,P) with

$$\epsilon \left(x,\psi ,P\right)∼\frac{P}{1+e^{-k\left(x-x_{0}\right)}}\cdot N\left(0,1\right).$$ (6)

This ensures that noise saturates to P as the stimulation strength becomes maximal and that the noise amplitude goes to zero as x gets smaller. Additionally, the noisy MEPs are clamped to have minimum 0, as peak-to-peak MEPs can only be positive. The MEP generation process is illustrated in Figure 1.

### 2.4 Motor mapping of synthetic and experimental data

For generating muscle representation maps in the motor cortex, we closely follow (Numssen et al., 2021; Weise et al., 2023). At step s of the sampling procedure, there are s | E | per ROI compartment in the sampled dataset. For each sample, we also have a corresponding MEP amplitude, that was either experimentally measured, or synthetically generated by the model from eq. 5. Then, for each compartment k, a sigmoid function is fitted to the MEP data using the Levenberg-Marquardt least-squared optimizer (Moré, 2006). The resulting $R^{2}$ values for all compartments are then combined to construct an $R^{2}$ map ${\text{R}}^{2}$ for the ROI (Numssen et al., 2021).

The fitting score ${\text{score}}_{f}$ is then computed as the inner product of a reference $R^{2}$ map ${\text{R}}_{ref}^{2}$, e.g. M or a high resolution map fitted with all samples in C, and an estimated $R^{2}$ map ${\hat{R}}^{2}$. The estimated map is computed based on a subset of samples, which may be selected using methods such as random sampling or FPS. Both maps are normalized to have $l^{2}$-norm 1:

$$score_{f}(R_{ref}^{2},{\hat{R}}^{2})=\frac{\langle R_{ref}^{2},{\hat{R}}^{2}\rangle }{||R_{ref}^{2}||\cdot ||{\hat{R}}^{2}}.$$ (7)

The score can be interpreted as the similarity or overlap of the two maps where a score of 1 is a perfect fit and 0 is the lower bound.

### 2.5 TMS experiment

#### 2.5.1 Ethics declarations

The Ethic Committee of the University of Freiburg Medical Center approved the investigation (application number: 23-1114-S1). All participants gave written informed consent before participation. We performed all experiments in accordance with relevant guidelines and regulations.

#### 2.5.2 Participants

Ten healthy, right-handed participants (6 females, 4 males; age [years]: mean ± std: 21.8±1.62 , range from 21 to 26) were recruited for the study. Participants had laterality index of mean ± std: 83.49±16.45 , range from 57.14 to 100, as determined by the Edinburgh Handedness Inventory (Oldfield, 1971). The selection process for volunteers began with a personal meeting with one of the study physicians, during which the exclusion and inclusion criteria were reviewed and approved. Next, participants who expressed interest in the study and met all inclusion criteria, while also not meeting any exclusion criteria as evaluated by the study neurologists, underwent structural MRI acquisition. The individual MRI images were then examined by the study radiologist to identify any brain pathologies. No participants showed signs of brain pathology and all of them were, therefore, eligible to be included in the study. In addition, we applied further exclusion criteria for data included in the analyses. One participant was excluded due to an incorrect search radius of 50 mm instead of the intended 30 mm (see Section 2.5.4 for details). Another participant was excluded due to delayed MEPs occurring outside the predefined time window of 18 to 35 ms following TMS application (Weise et al., 2020). Therefore, of the 10 volunteers, data from eight were used in the motor mapping analysis.

#### 2.5.3 MRI

High resolution MRI data were acquired with a 3-Tesla SIEMENS MAGNETOM Prisma scanner and a 64-channel head coil. T1-weighted images were acquired with the following parameters: sequence: Magnetization Prepared - RApid Gradient Echo (MPRAGE); voxel size: 1.0 × 1.0 × 1.0 mm; TR: 2500 ms; TE: 2.82 ms; flip angle: 7∘; FoV: 256 mm; fat suppression: water excitation; orientation: sagittal; no. of slices: 192; total acquisition time: 3 min. 58 s. T2-weighted images were acquired with the following parameters: sequence: Sampling Perfection with Application optimized Contrasts using different flip-angle Evolutions (SPACE); voxel size: 1.0 × 1.0 × 1.0 mm; TR: 2500 ms; TE: 231 ms; FoV: 256 mm; fat suppression: no; orientation: transversal; no. of slices: 160; total acquisition time: 6 min. 42 s. In addition to the high-resolution 3D anatomical images, we also acquired diffusion tensor imaging data with the following parameters: voxel size: 1.5 × 1.5 × 3.0 mm; TR: 2800 ms; TE: 88 ms; no. of directions: 65; FoV: 222 mm; total acquisition time: 6 min. 22 s.

#### 2.5.4 Head modeling and macroscopic E-field simulations

We used an open-access toolbox called Simulation of Non-invasive Brain Stimulation (SimNIBS; version 3.2.6) to create head models and perform E-field simulations (Thielscher et al., 2015). Anatomically realistic multi-compartment head models were created by calling the *headreco* pipeline using individual T1- and T2-weighted anatomical and diffusion-weighted MRI sequences. To enhance the smoothness of the skin compartment, we individually tailored the number of cortical smoothing repetitions, ranging between 100 and 200 repetitions (Turi et al., 2022). The final head mesh contained around 4 million tetrahedra. For the following tissue compartments, we used the default isotropic conductivity values [in S/m]: eyes (0.5), scalp (0.465), bone (0.01), cerebrospinal fluid (1.654), whereas for the gray matter and white matter compartments, we assigned volume normalized anisotropic conductivities (Opitz et al., 2011). E-field calculations for each stimulation were conducted with high resolution anisotropic finite element models. For all simulations, we set the stimulation intensity at a coil-current rate of change of 1.49 A/µs that corresponded to 1% MSO for our TMS device. The scalp-to-coil distance was determined individually by measuring hair thickness using a depth gauge (Beynel et al., 2020; Dannhauer et al., 2022). Additionally, an extra 1 mm was added to the scalp-to-coil distance to account for the touch sensor of the TMS coil. We established the ROI center based on a prior meta-analysis (Mayka et al., 2006). Specifically, we converted the primary motor cortex coordinates from the Montreal Neurological Institute coordinate space (MNI152; x-37, y-21, z+58) into the subject-space. The primary coordinate was subject to individual visual inspection, and where necessary, adjustments were made to ensure its localization within the precentral gyrus. Then, we calculated the Euclidean distance between the individual FDI coordinate within the gray matter compartment and the scalp compartment, choosing the scalp position with the closest proximity as the stimulation target. In the E-field simulations, we employed a search radius of 30 mm around the ROI center, positioning coil locations with a spatial resolution of 3 mm. For each cortical target, we systematically adjusted the coil’s rotation angle in increments of 5° from 0° to 175°. We excluded orientations at and beyond 180° since they predominantly affected the E-field direction. The constraints on these parameters are based on extensive preliminary simulations, as described in Supplementary Appendix A. We extracted the element-wise | E | from the middle gray matter compartment using a cylindrical ROI with a radius of 20 mm from the ROI center (Numssen et al., 2021). Therefore, we utilized two radii around the ROI center. One radius, set at 30 mm, defined the coil search radius, while the other radius corresponded to the actual ROI used for analyzing the | E | . A larger radius was applied for the coil search to further enhance differences in the | E | distributions within the ROI, particularly between the center and edge regions.

#### 2.5.5 Neuronavigated robotic-arm-guided TMS

We employed a neuronavigated, robotic-arm-guided TMS system in the experimental phase. Single biphasic TMS pulses were administered through a MagPro X100 stimulator (MagVenture, firmware version: 7.2.4) using a robot-compatible Cool-B65 CO figure-of-eight-coil. The Localite neuronavigation system (TMS Navigator Robotic Edition Axilum cobot; version 3.3.8) and an Axilum Cobot were employed for precise coil guidance (version 1.3.2.0; controller version 2.1.10.148). Matsimnibs files, corresponding to individual E-field simulations and containing the 4-D affine transformation matrices that define coil positions and directions, were converted to HTML files compatible with the neuronavigation system. These files were then uploaded to the neuronavigation software using Localite’s Session Utility Tool (version 3.3.15). The coil position selection was controlled by the data collector by using Localite’s neuronavigation software.

MEPs were recorded from the participants’ right FDI muscle. We placed one self-adhesive surface electrode over the FDI muscle belly and another one at the proximal interphalangeal joint (Kendall, H124SG, Cardinal Health Germany 507 GmbH, Norderstedt, Germany). The surface electrodes were connected to the amplifier system (D-360, Digitimer Ltd., UK, Welwyn Garden City) with bandpass filtering ranging from 10 Hz to 2 kHz. The amplifier system was subsequently connected to a Hum Bug Noise Eliminator (Digitimer Ltd., UK, Welwyn Garden City), which was then linked to a data acquisition interface (Micro 1401-4, CED Ltd., Cambridge, UK) with a sampling rate of 5 kHz. Participants wore earplugs with a single-number rating isolation value of 35 dB during the experiment to attenuate the click sound generated by TMS (Moldex Spark Plugs 7800, Walddorfhäslach, Germany).

The synchronization between the TMS device and EMG data acquisition system was accomplished through a custom MATLAB application developed in-house. For controlling the time and the intensity of stimulation, we utilized the MAGIC toolbox (Saatlou et al., 2018). Raw EMG data from the data acquisition unit was directly accessed using the “Matced” interface (available at https://ced.co.uk/downloads/contributed). All incoming data was stored by the MATLAB application irrespective of whether a trial was later included or excluded from analysis. To ensure the integrity and reliability of the study, trials were not subject to deletion during online data collection. Trials were automatically excluded (but not deleted) from the data analysis if they displayed pre-stimulus muscle activity with a peak-to-peak amplitude exceeding 50 μV within a 100 ms time-window before the application of TMS. The peak-to-peak amplitude of MEPs were automatically extracted from 18 to 35 ms time window after the TMS pulse (Weise et al., 2023). In the resting motor threshold (RMT) estimation, we used a minimum peak-to-peak amplitude of 50 μV. Together with the neuronavigated TMS-cobot, our MATLAB application reduces reliance on operator discretion for subjective decisions related to free-hand coil positioning, stimulation intensity determination, and online trial exclusions under limited time. Consequently, our systematic approach ensures an accurate and reproducible experimental workflow, prioritizing both participant safety and research integrity.

RMT was determined utilizing a maximum likelihood estimation based adaptive threshold hunting algorithm, following the recommendations of the International Federation of Clinical Neurophysiology (Rossini et al., 2015). This algorithm employs parameter estimation through sequential testing, commonly referred to as the PEST method (Pentland, 1980). Initially described by Awiszus (2003), it was subsequently implemented as desktop software known as the Motor Threshold Assessment Tool (MTAT; version 2) (Awiszus & Borckardt, 2011) (but see references (Koponen & Peterchev, 2022) and (Borckardt, 2022) for a recent update of MTAT to version 2.1). The current study utilized the MATLAB version of MTAT, obtained through personal communication with Julkunen (2019). The algorithm yields an accurate estimation of the RMT within 13 to 30 trials. The convergence of the RMT estimate, along with the estimated threshold and the corresponding MEPs, was verified for each estimation procedure by the data collector.

During motor mapping, each participant was planned to receive a total of 300 stimulations, consisting of 150 coil configurations selected using random sampling and 150 using the FPS method. However, the actual number of stimulations was slightly higher due to the need to repeat trials caused by muscle preactivity. The random and FPS stimulations were further divided into blocks of 50 TMS trials each. The order of random and FPS blocks was counterbalanced across participants: five participants started with the random blocks and finished with the FPS blocks, and vice versa. Before assigning the FPS trials to the three TMS blocks of 50 TMS trials each, the order of the FPS trials were randomized. This step was not necessary for the TMS trials generated by the random method. Motor mapping was performed at 120% of the RMT. However, for two participants, a reduced intensity of 110% RMT was used to minimize stimulation-induced subjective discomfort.

## 3 Results

### 3.1 Motor mapping on synthetic data

**Fig. 2.**
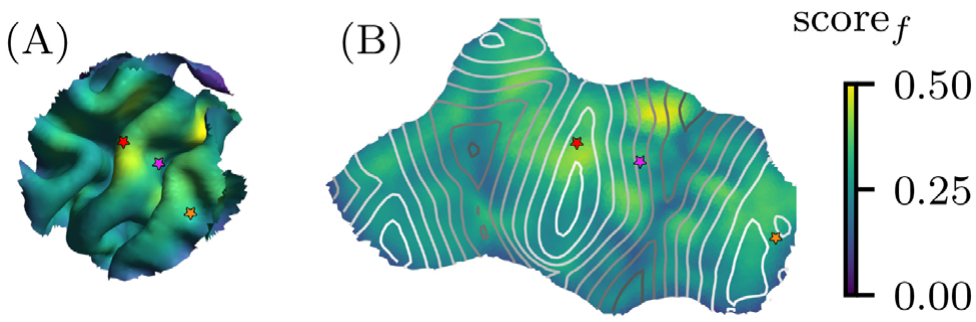
Optimal fitting scores depend on location of muscle representation. We placed the muscle representation at every possible element in the ROI and tested how well the ground-truth motor map could be recovered with a fitted $R^{2}$-map from noisy MEP simulations, computing ${\text{score}}_{f}(\text{M},{\hat{\text{R}}}^{2})$ for each location. (A) The scores mapped to a three-dimensional representation of the ROI. (B) The three dimensional ROI surface was flattened with multidimensional scaling to obtain a two dimensional representation based on the pairwise matrix of geodesic distances between ROI compartments. The contour lines indicate sulcal depth, with light colors corresponding to the gyral crown and darker colors to the sulcal fold. The colored stars mark identical locations in both ROI representations.

First, we tested how well a ground truth motor map could be estimated depending on the location of the muscle representation. To do this, we placed $k_{max}$ of the MGM M at every possible compartment k of the ROI, generated noisy MEP amplitudes for a dense grid of 2,853 coil configurations (one per configuration), and computed ${\hat{R}}^{2}$ based on the respective |E| and MEP amplitudes. Next, we calculated the overlap score, ${\text{score}}_{f}(\text{M},{\hat{\text{R}}}^{2})$ , for each compartment. Figure 2 shows that there is no clear trend when comparing locations on the gyral crown or lip to those on the sulcal wall. Only locations within the gyral fold exhibit consistently low fitting scores across the entire ROI.

To evaluate the efficacy of sampling methods for motor mapping, we compared their ability to fit motor maps to noisy synthetic MEP amplitudes generated from predefined motor maps M. The MEP amplitudes were generated as described in Section 2.3. The experiment was run on a single head model and repeated using 10 sampling seeds and 10 seeds for generating MEP noise, resulting in a total of 100 runs per parameter setting for both FPS and random sampling.

**Fig. 3.**
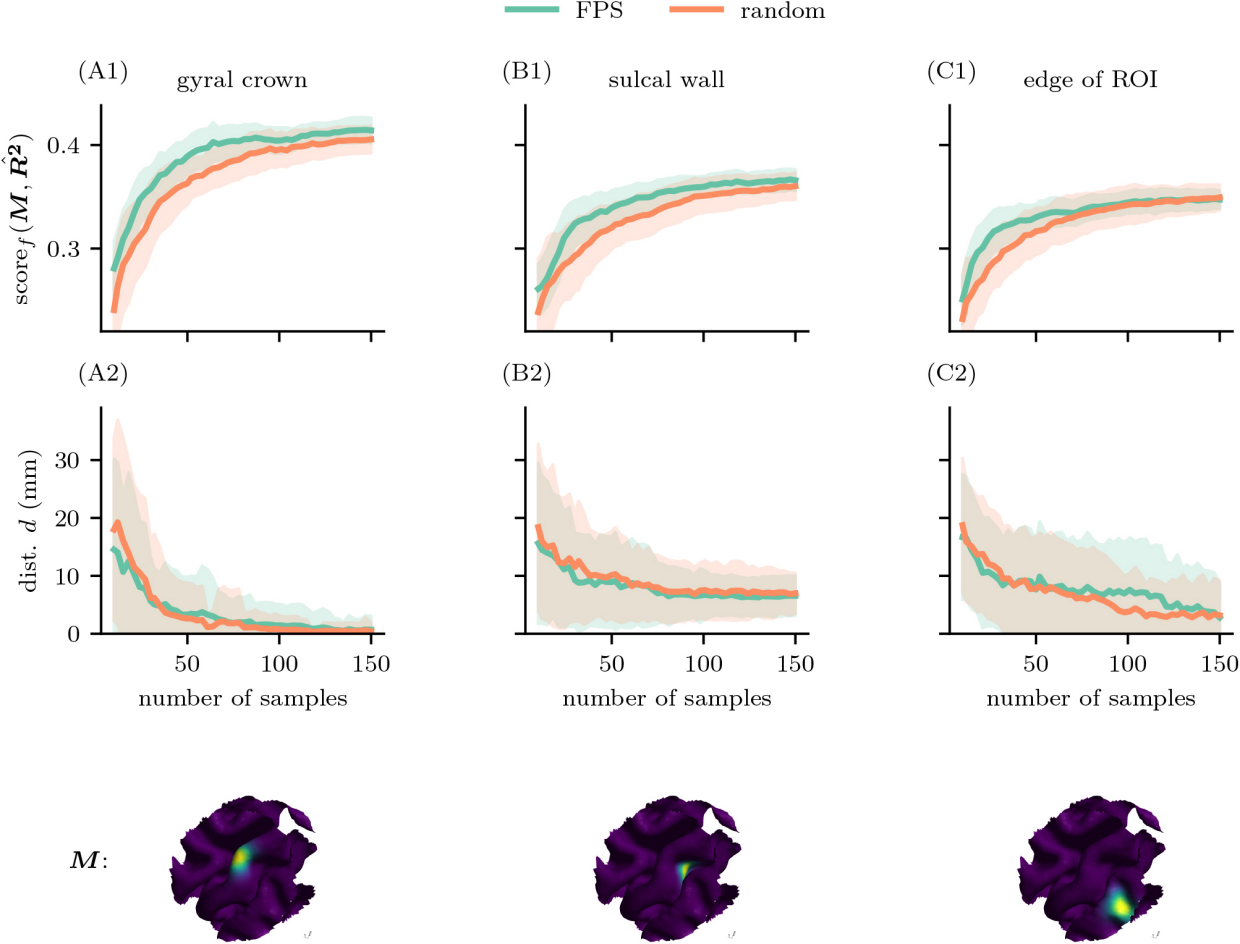
Subsampling from the synthetic data demonstrates improved sample-efficiency of the FPS algorithm compared to the random sampling across all simulation conditions. We subsampled from a large dataset of simulated |E|. The motor maps M used to generate noisy MEPs were positioned at three locations: the gyral crown at the center of the ROI (A), the sulcal wall at the center of the ROI (B), and the gyral crown at the edge of the ROI (C). (A1-C1) We computed ${\text{score}}_{f}(\text{M},{\hat{\text{R}}}^{2})$ as a function of the number of samples. (A2-C2) Additionally, we measured the geodesic distance d between the peak location of M and the peak of the estimated $R^{2}$ map, ${\hat{\text{R}}}^{2}$. Lines indicate the mean across 100 runs, and shaded areas represent the standard deviation. The corresponding fictive muscle representations M are shown below each plot. FPS outperforms random sampling for all three simulated motor maps in terms of ${\text{score}}_{f}$.

**Fig. 4.**
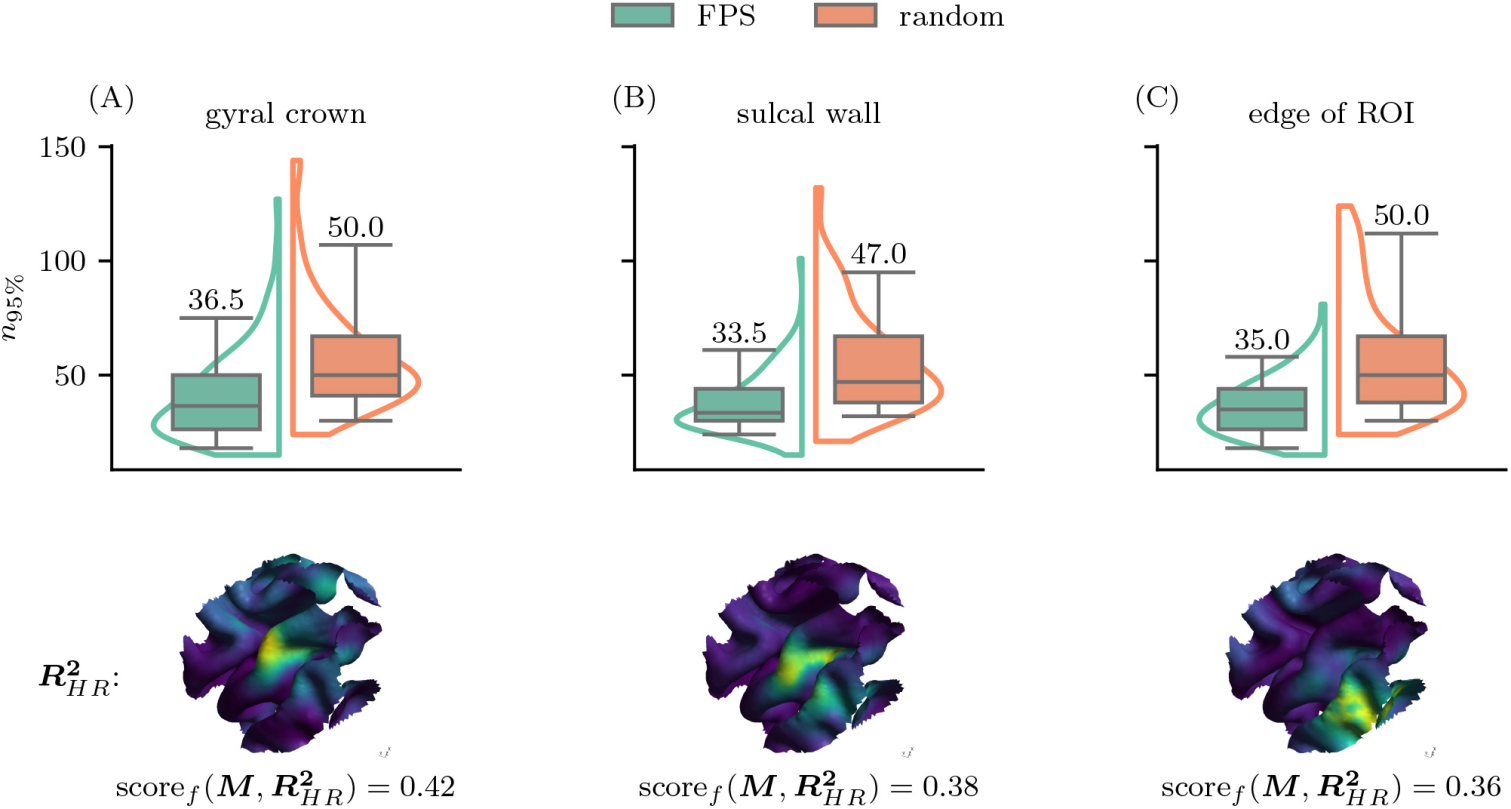
FPS subsampling achieves optimal fit with fewer samples. We subsampled from a large dataset of simulated |E|, using motor maps M positioned at the gyral crown in the center of the ROI (A), the sulcal wall in the center (B), and the gyral crown at the edge (C). We assessed how many samples were needed to reach 95% overlap with the high-resolution map ${\text{R}}_{HR}^{2}$, defined as ${\text{score}}_{f}({\text{R}}_{HR}^{2},{\hat{\text{R}}}^{2})>0.95$ . Each box summarizes 100 random initializations: the center line shows the median (which is also provided numerically directly above the maximum whisker of each boxplot), whiskers span from the 5th to the 95th percentile, and violins show a fitted kernel density estimate of the distribution. The score shown below each ${\text{R}}_{HR}^{2}$ map indicates its overlap with the corresponding ground truth M. FPS consistently requires fewer samples than random sampling for all three simulated muscle representations.

Figure 3A1-C1 show that the ground truth MGM M is recovered substantially faster with FPS compared to random sampling. While both methods converge to similar scores for large sample sizes, FPS is more data efficient in the early sampling stages. Overall, the scores indicate that muscle representations located at the ROI center identified with higher precision compared to those at the ROI edge. The same can be observed for the target being at gyral crown compared to the sulcal wall or fold. However, even at the ROI edge, target localization was feasible, particularly with FPS-guided stimulation, underscoring the robustness of FPS for motor mapping. Regarding the geodesic distance d between the peaks of M and ${\hat{\text{R}}}^{2}$, we observe no significant difference between the methods (Fig. 3A2–C2). These distances converge toward zero primarily when M is located on a gyral crown (Numssen et al., 2021). Larger residual distances are more frequent when the motor representation lies in regions where TMS induces substantially weaker |E|, such as deep sulcal walls and fundi. In these cases, the distributions of induced | E | lack sufficient focality to clearly distinguish adjacent regions. Additionally, $R^{2}$ maps inherently reflect correlation strength and may highlight regions with strong but potentially indirect associations with the MEP signal. For example, in Figure 4B, the fitted $R^{2}$ map reveals a broad area of elevated $R^{2}$ values, peaking across both the crown and sulcal wall. This underscores the limitation of using $R^{2}$ maps as definitive indicators of excitability. To establish an upper performance limit, we created a high resolution (HR) reference map ${\text{R}}_{HR}^{2}$ with ≈13000 MEP samples. This HR map represents the theoretical outcome if stimulation were applied to every point on a dense grid (spatial resolution 2 mm, angular resolution 10∘, search radius of 30 mm) and served as the ground truth target. The performance of each sampling method was quantified by determining the number of samples required to achieve a fitting score ${\text{score}}_{f}({\text{R}}_{HR}^{2},{\hat{\text{R}}}^{2})$ of 0.95 , corresponding to 95% overlap with ${\text{R}}_{HR}^{2}$. Figure 4 confirms that the ${\text{R}}_{HR}^{2}$ maps were recovered more quickly and accurately using FPS compared to random sampling across all three tested simulated muscle representations (see also Supplementary Appendix C). When the target was located at the center of the ROI on the gyral crown, FPS achieves superior performance, requiring an average of 40.39±19.96 (mean ± std) samples, compared to random sampling 60.64±25.00 . This results in a difference of ≈20 samples (Fig. 4A). For a target at the center of the ROI but on the sulcal wall, the difference between the methods narrowed, with FPS requiring 37.42±13.30 samples compared to 55.20  ±  22.50 for the random sampling, a reduction of ≈  18 samples (Fig. 4B). At the edge of the ROI, FPS demonstrates the largest improvement, requiring 35.98  ±  12.87 compared to 59.15  ±  24.61 for the random sampling, a reduction of almost 24 samples (Fig. 4C).

On average, FPS required approximately 40 samples to achieve a fitting score of 95% with the ${\text{R}}_{HR}^{2}$ map across all target locations. These results underscore the significant sample efficiency of FPS for motor mapping. Furthermore, FPS demonstrated reduced sensitivity to randomization of initialization, as indicated by smaller whisker extensions and lower standard deviations in Figure 4.

Importantly, FPS consistently outperformed random sampling regardless of MEP model parameters ψ. A comprehensive parameter sweep over MGM standard deviation σ, MEP generation noise P, and sigmoid slope k yielded comparable performance differences. For larger noise amplitudes P the scores generally dropped. The same was observed for smaller MGM standard deviations, equivalent to more focalized muscle representations. The change of the sigmoid slope k had no substantial effect on the results. These observations are described and visualized in more detail, and numerical values of P and the model parameters ψ are provided in Supplementary Appendix B.

### 3.2 Motor mapping on human participants

Building on the insights gained from synthetic data, we next evaluated the performance of the proposed sampling methods in motor mapping experiments conducted with human participants to assess their practical applicability and robustness.

No serious adverse effects (e.g., epileptic seizure, syncope, etc.) occurred during or after the experiment. Among the 10 participants, none reported perceiving phosphenes during the experiment. One participant experienced a mild headache during the experimental session, rated at the lowest intensity (1/10) on a Likert scale. Overall, participants described the stimulation pulses as a moderate tactile sensation on the scalp, with a mean score of 4.75±2.15 on a 10-point Likert scale (1 = minimal sensation, 10 = maximal sensation). For one participant with a mapping intensity at 79% MSO, the stimulation was perceived as uncomfortably intense at specific frontal and temporal locations. Participants also described the stimulation pulses as moderate auditory sensations with a mean score of 4.55±2.54 on a 10-point Likert scale.

**Fig. 5.**
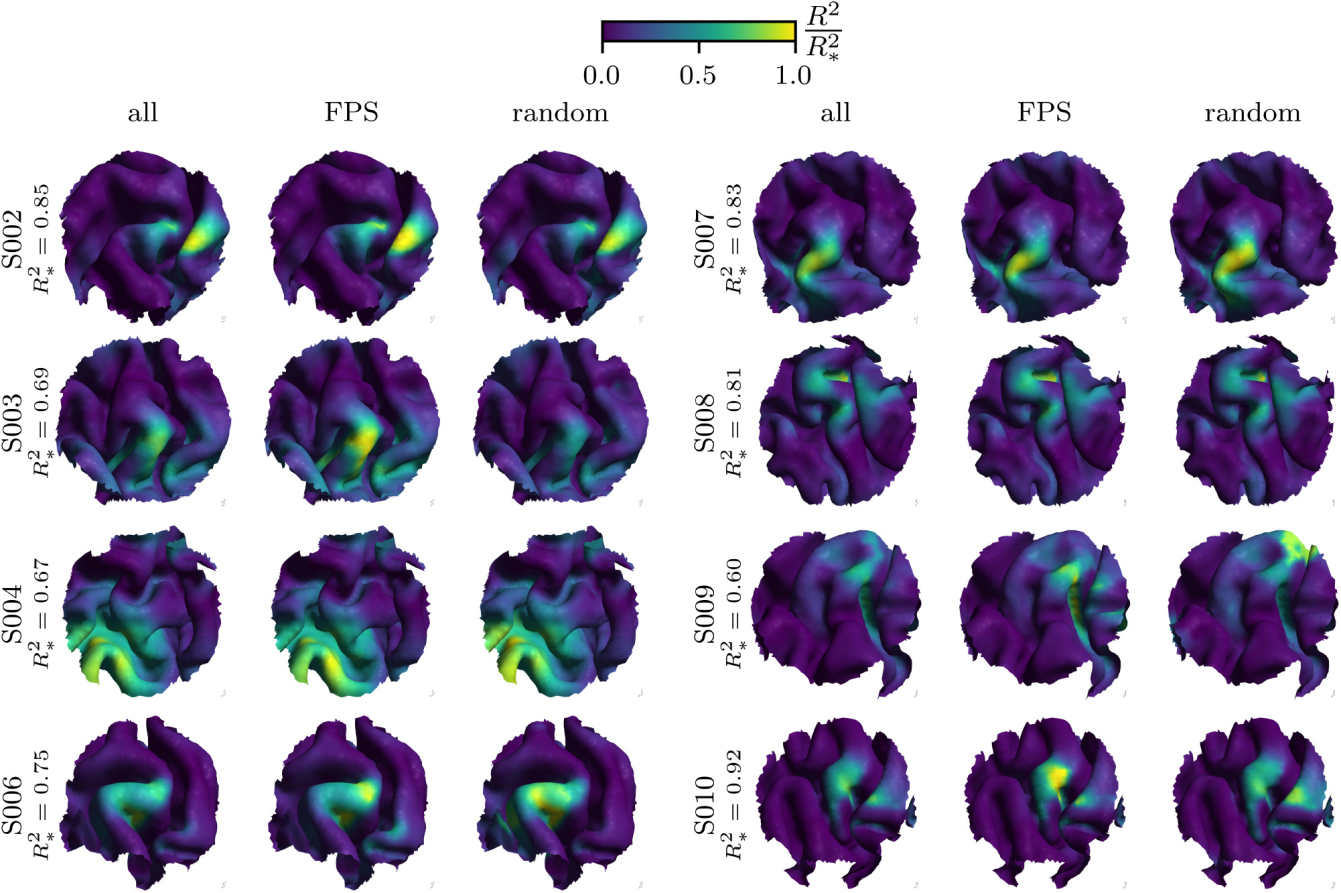
Predicted FDI motor cortical representations of all participants. For each participant that entered the analysis, the results given all samples, FPS samples, and random samples are depicted in the three columns. The $R^{2}$-values are normalized by the subject-specific maximum value $R_{\text{*}}^{2}$. Theses maximal values range from 0.60 to 0.92. The $R^{2}$ maps can be interpreted as the subject-specific muscle representation of the FDI muscle. The representation for the FDI muscle of most participants lies at the gyral crown of the precentral gyrus.

For each participant, we generated a target map ${\hat{\text{R}}}_{all}^{2}$, by fitting all measurements, as well as $R^{2}$-maps based on data from the random and FPS block. These maps are shown in Figure 5. The MNI coordinates for the FDI muscle representations (see in Supplementary Appendix E) are comparable to those reported in previous TMS studies, e.g., (Bungert et al., 2017; Kim et al., 2021; Numssen et al., 2021). Subsets of the two blocks were then analyzed to determine how many stimulations were required to approximate the target map. Two metrics were evaluated: (1) the fitting score ${\text{score}}_{f}({\hat{\text{R}}}_{all}^{2},{\hat{\text{R}}}^{2})$ between the target map and subset maps, as defined in Equation 7, which the similarity muscle representation distributions across the ROI surface; and (2) the geodesic distance d between the peak $R^{2}$ value of the target map and the subset map, representing the distance between the muscle representation centers.

**Fig. 6.**
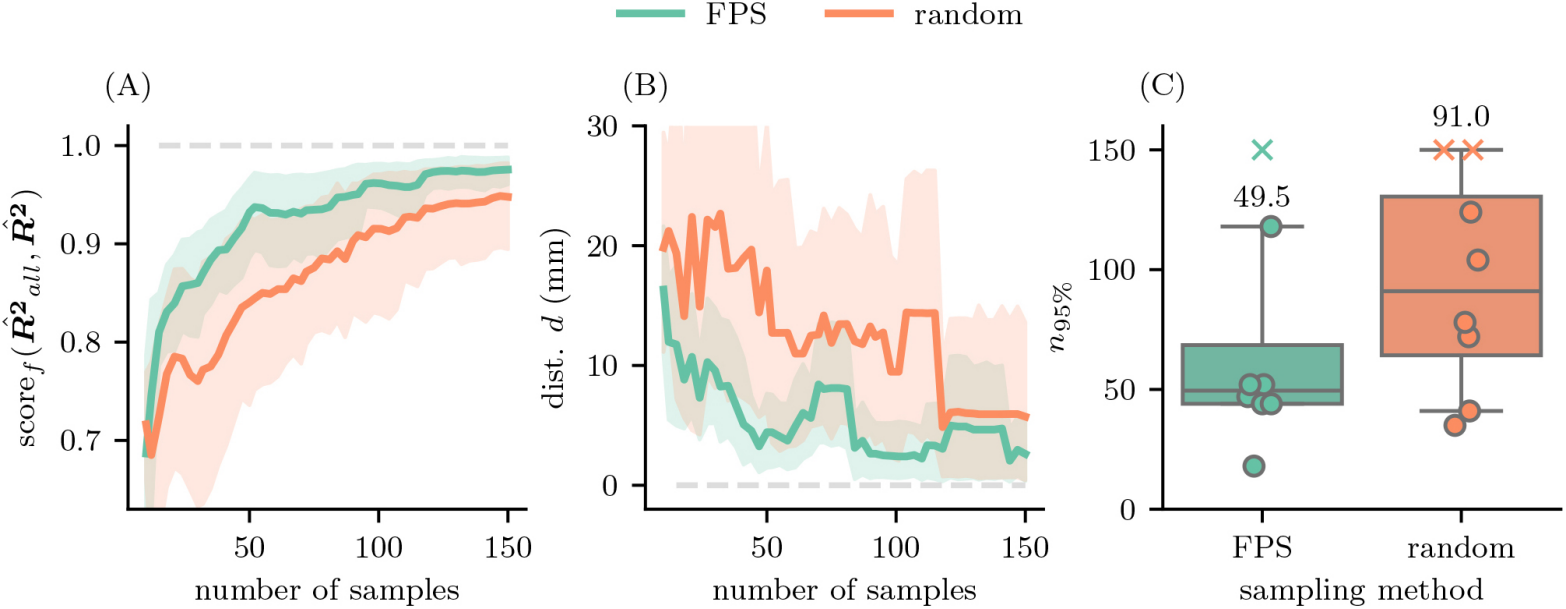
Subsampling from the experimental dataset. For every other sample we calculated (A) the fitting score ${\text{score}}_{f}({\hat{\text{R}}}_{all}^{2},{\hat{\text{R}}}^{2})$ as overlap of the fitted motor maps ${\hat{\text{R}}}^{2}$ with the $R^{2}$ map fitted with all valid samples ${\hat{\text{R}}}_{all}^{2}$ (ideally 300), and (B) the geodesic distance *d* of the respective mesh elements with the highest $R^{2}$-values. The lines represent the mean across participants and the shaded area the 95% confidence interval. (C) We tested how many samples were needed to reach 95% overlap with the target $R^{2}$ map (${\text{score}}_{f}>0.95$ ). The boxes summarize $n_{95\text{\%}}$ across participants and extend from the 25th percentile to the 75th percentile. The line in the boxes represents the median (which is also provided numerically directly above the maximum whisker of each boxplot), the whiskers extend from the 5th to the 95th percentile, the points represent single participants, and participants where ${\text{score}}_{f}$ did not reach 95% are marked as crosses and placed at $n_{95\text{\%}}=150$ . FPS outperforms random sampling especially in the early sampling stage and is approaching an optimal overlap and small target distance much faster.

Figure 6 illustrates several key differences between FPS-based stimulation maps and random sampling methods stimulation maps. FPS-derived maps converged to the target map notably faster, with this advantage being most pronounced during the early stages of sampling (Fig. 6A). The geodesic distance d between the highest $R^{2}$ value of each sampled map and the target map was consistently smaller for FPS compared to random sampling, particularly at lower sample sizes (Fig. 6B). Moreover, both ${\text{score}}_{f}({\hat{\text{R}}}_{all}^{2},{\hat{\text{R}}}^{2})$ and the distance d exhibit greater robustness across participants in FPS-derived maps, as reflected by narrower 95% confidence intervals for these metrics. Notably, FPS achieved 95% overlap with the target map using ≈ 50 E-field MEP pairs, whereas random sampling required nearly twice as many samples to reach the same level of accuracy (Fig. 6C). To ensure generalizability of our findings, we conceptually replicated the analysis using an independent dataset previously published by Numssen et al. (2021), as described in Supplementary Appendix D.

## 4 Discussion

The current state-of-the-art approach to TMS-based motor mapping relies on the random selection of coil configurations during MEP assessments, followed by retrospective modeling of the induced E-field (Numssen et al., 2021; Weise et al., 2020, 2023). This method has significantly advanced the field and remains appealing due to its simplicity and low demands on research infrastructure and prospective computations, as it avoids the need for a TMS robot and prospective E-field simulations. However, it typically requires a large number of stimuli to produce accurate spatial representations. This represents a crucial limitation, as both research and clinical applications of TMS would greatly benefit from more sample-efficient motor mapping methods. In this study, we introduced a novel approach that prospectively selects coil configurations based on the characteristics of their | E | . Our findings demonstrate that this method can substantially reduce the number of trials required for motor mapping while maintaining high spatial accuracy.

The primary results of our study demonstrate that the FPS algorithm facilitates more sample-efficient motor mapping compared to random sampling. FPS achieved the same accuracy level (95% overlap with the target map) with approximately half the number of stimulation trials (med. 49.5 trials) required by random sampling (med. 91 trials; see also Fig. 6C). Furthermore, FPS exhibited greater robustness across participants, as reflected by substantially narrower confidence intervals in both fitting scores and geodesic distances (see Fig. 6A-B).

In synthetic data analyses, the FPS algorithm also consistently outperformed random sampling. In all scenarios tested, including muscle representations located at the gyral crown, sulcal wall, and ROI edges, FPS demonstrated superior sampling-efficiency (Fig. 4A-C). The advantage of FPS was especially pronounced for representations located at the sulcal wall and ROI edge (see Fig. 3A1-C1). Importantly, the superior sample-efficacy was not achieved at the expense of spatial accuracy, since the geodesic distance d between the true and estimated loci were closely matched for the FPS and random methods (see Fig. 3A2-C2). Taken together, we conclude that the synthetic and experimental findings congruently underscore the superior sampling-efficiency of the FPS method.

Reducing the number of required trials alleviates participant burden, which is especially important in clinical settings. Although the FPS method can substantially reduce the total number of required trials, this advantage is limited by the current implementation, which involves relatively long inter-trial intervals. These intervals are necessary to verify the order of prospectively defined trials within both the neuronavigation system and our MEP assessment tool, requiring approximately 12 s per trial on average. Unfortunately, automatic trial selection is not yet supported in the CE-certified version of the neuronavigation software. In contrast, with the random method, a well-trained and experienced operator can achieve coil placement at randomly selected locations in under 5 s. However, manual targeting of specific neuronavigated sites results in substantially longer placement times than those achieved using a robotic arm guided by neuronavigation.

Another finding from our synthetic data analyses is that muscle representations can be recovered with comparable efficacy in both the gyral crown and sulcal wall regions (see Fig. 2). This is particularly relevant given the ongoing debate regarding the precise loci of initial TMS activation. While some studies suggest that activation primarily occurs at the gyral crown, others propose the sulcal wall as the initial target (Bungert et al., 2017; Fox et al., 2004; Gomez–Tames et al., 2020; Krieg et al., 2015; Laakso et al., 2018). Our simulations revealed that although motor representation recovery is less optimal within deeper cortical folds, the mapping method is unbiased toward either the gyral crown or sulcal wall and can recover representations equally well in both regions. However, it is important to note that due to variations in coil-to-cortex distance arising from cortical folding, FPS is inherently biased toward selecting samples with high | E | , and thus tends to favor gyral crown regions (see limitations below). As a result, the experimental finding that the most probable motor representations for the FDI muscle are located in the gyral crown and lip regions should be interpreted in the context of this methodological bias (see Fig. 5). Nevertheless, further addressing the inherent bias of the FPS method in future studies could ultimately enhance the spatial precision of motor mapping.

A notable strength of the FPS method is its computational simplicity. The algorithm does not require specialized expertise, operates on standard CPU infrastructure without GPU acceleration, and has a computation time in the range of seconds. It is important to note that the E-field simulations are a prerequisite for any E-field-based mapping approach and are not specific to FPS. While the E-field simulations in the random sampling approach are performed retrospectively, FPS requires them to be performed prospectively to guide coil placement. This simulation step typically takes a few hours. These features could render FPS accessible to a broad range of research and clinical settings, assuming a stable and professionally maintained implementation. The method is particularly beneficial for preoperative functional cortical mapping in patients (Haddad et al., 2021; Jeltema et al., 2021; Krings et al., 1997, 2001; Muir et al., 2022; Picht et al., 2011; Raffa et al., 2019; Sartori et al., 2023; Sollmann et al., 2016; Umana et al., 2021) and for exploring higher-order cortical functions in cognitive neuroscience studies involving healthy participants (Jing et al., 2023). In these applications, streamlining mapping approaches that reduce participation time while maintaining data quality are essential and may, for example, enable functional mapping of complex cognitive processes such as internal world models (Diester et al., 2024).

An additional strength of our novel method lies in its ability to minimize operator bias when implementing a truly random randomization strategy. Humans typically struggle to generate true randomness; as a result, random sampling in real experimental conditions often introduces biases, such as a tendency to revisit previously selected sites. However, in our study, this bias was effectively mitigated through the use of algorithmically generated random sampling combined with a neuronavigated robotic arm. These findings suggest that the differences between FPS-based and human-based random sampling might be even more pronounced in real-world experimental settings.

Despite these advantages, our novel approach is not without limitations. The FPS method is biased towards maximizing | E | differences in gyral crown regions. Due to variations in coil-to-cortex distance resulting from the cortical folding pattern, | E | are typically higher at gyral crowns—where the coil is closer to the cortex—than in sulcal walls or fundi, where the greater distance leads to weaker fields. Consequently, when FPS seeks to maximize the minimum distance between | E | maps, it is more likely to find large dissimilarities near the gyral crowns. This limitation is specific to the $R^{2}$-method and is not confined to our particular experiment (e.g., (Numssen et al., 2021)). However, the distribution of | E | in the cortex exhibits spatial correlations, as regions with high | E | in the gyral crown often extend into adjacent areas, such as the sulcal walls. As a result, selecting points with high dissimilarity at the crown may still indirectly promote diverse sampling, including deeper regions of the sulcal walls. Nevertheless, developing a sampling algorithm that explicitly accounts for these anatomical differences e.g., by applying location-weighted distance metrics based on cortical-folding morphology, remains an important direction for future work.

Additionally, the proposed approach relies on neuronavigated, robotic-arm-guided systems to implement pre-generated coil configurations. Without robotic assistance, experimental validation becomes prohibitively time-consuming. In contrast, random sampling can be conducted with neuronavigation alone, without requiring TMS robot infrastructure or prospective E-field modeling. Furthermore, the FPS method still requires programming expertise in Python or MATLAB, which currently limits its applicability in routine clinical settings. Moreover, FPS introduces an additional computational burden, as it requires calculating the E fields for all candidate coil configurations (C). In contrast, random sampling requires calculations only for the coil configurations that were actually utilized. Nonetheless, the computational overhead of the FPS method is somewhat offset by the greater number of stimulation trials typically required by the random method to achieve a comparable level of mapping precision.

Furthermore, the FPS method currently depends on pre-generated coil configurations, limiting its flexibility to navigate to dynamically generated target locations during motor mapping. Future work should explore closed-loop mapping approaches, where coil configurations are iteratively updated online based on prior stimulation outcomes. The main difficulty of such an approach would be an efficient implementation of the simulation of the E-field that is fast enough to be run during online stimulation to inform the next coil configuration (c.f. Hasan et al., 2025). However, such advancement could further optimize mapping efficiency and adaptability significantly.

Additionally, the current study utilized $R^{2}$-maps to evaluate the goodness of fit; however, this metric may face challenges in distinguishing between closely situated candidate regions. This limitation arises from its inherent assumption of fitting a sigmoid to a single compartment, presuming that a single anatomically restricted location underlies the causal relationship between | E | and response strength. Consequently, it disregards the possibility of multi-modal or combinatorial effects. This may lead to a spatially inconclusive motor mapping outcome, even when all trials from both the random and FPS conditions are included (e.g., see motor mapping results for participant 6 in Fig. 5). Incorporating more advanced statistical approaches could improve the specificity and robustness of the mapping process. Regardless of these consideration, our findings highlight the potential of the FPS algorithm to significantly enhance the efficiency and robustness of TMS-based motor mapping. By reducing trial counts, improving consistency, and maintaining high spatial accuracy, FPS represents a promising step forward for both clinical and research applications of TMS.

## Supplementary Material

Supplementary Material

## Data Availability

The code for conducting the E-field modeling, simulations and analyses in the present study is available for download at the following Github repository https://github.com/david-schu/efield-informed-motor-mapping. The raw datasets supporting this study are available from the corresponding author upon reasonable request. Data generated during the simulations were not archived due to their substantial size.

## References

[IMAG.a.1056-b1] Allen, E. A., Pasley, B. N., Duong, T., & Freeman, R. D. (2007). Transcranial magnetic stimulation elicits coupled neural and hemodynamic consequences. Science, 317(5846), 1918–1921. 10.1126/science.1146426 17901333

[IMAG.a.1056-b2] Awiszus, F. (2003). TMS and threshold hunting. In Supplements to clinical neurophysiology (pp. 13–23). Elsevier. 10.1016/s1567-424x(09)70205-3 14677378

[IMAG.a.1056-b3] Awiszus, F., & Borckardt, J. (2011). Tms motor threshold assessment tool (mtat 2.0). Brain Stimulation Laboratory, Medical University of South Carolina, USA. 10.1016/j.brs.2010.10.001

[IMAG.a.1056-b4] Barker, A. T., Jalinous, R., & Freeston, I. L. (1985). Non-invasive magnetic stimulation of human motor cortex. The Lancet, 325(8437), 1106–1107. 10.1016/s0140-6736(85)92413-4 2860322

[IMAG.a.1056-b5] Batail, J.-M., Xiao, X., Azeez, A., Tischler, C., Kratter, I. H., Bishop, J. H., Saggar, M., & Williams, N. R. (2023). Network effects of stanford neuromodulation therapy (SNT) in treatment-resistant major depressive disorder: A randomized, controlled trial. Translational Psychiatry, 13(1), 240. 10.1038/s41398-023-02537-9 37400432 PMC10318050

[IMAG.a.1056-b6] Beynel, L., Davis, S. W., Crowell, C. A., Dannhauer, M., Lim, W., Palmer, H., Hilbig, S. A., Brito, A., Hile, C., Luber, B., Lisanby, S. H., Peterchev, A. V., Cabeza, R., & Appelbaum, L. G. (2020). Site-specific effects of online rTMS during a working memory task in healthy older adults. Brain Sciences, 10(5), 255. 10.3390/brainsci10050255 32349366 PMC7287855

[IMAG.a.1056-b7] Bijsterbosch, J. D., Barker, A. T., Lee, K.-H., & Woodruff, P. W. (2012). Where does transcranial magnetic stimulation (TMS) stimulate? modelling of induced field maps for some common cortical and cerebellar targets. Medical & Biological Engineering & Computing, 50, 671–681. 10.1007/s11517-012-0922-8 22678596

[IMAG.a.1056-b8] Borckardt, J. J. (2022). Preventing misestimation of transcranial magnetic stimulation motor threshold with mtat 2.0: A response to Koponen and Peterchev. Brain Stimulation, 15(5), 1321. 10.1016/j.brs.2022.08.021 36180038

[IMAG.a.1056-b9] Bungert, A., Antunes, A., Espenhahn, S., & Thielscher, A. (2017). Where does TMS stimulate the motor cortex? combining electrophysiological measurements and realistic field estimates to reveal the affected cortex position. Cerebral Cortex, 27(11), 5083–5094. 10.1093/cercor/bhw292 27664963

[IMAG.a.1056-b10] Castrillon, G., Sollmann, N., Kurcyus, K., Razi, A., Krieg, S. M., & Riedl, V. (2020). The physiological effects of noninvasive brain stimulation fundamentally differ across the human cortex. Science Advances, 6(5), eaay2739. 10.1126/sciadv.aay2739 32064344 PMC6994208

[IMAG.a.1056-b11] Classen, J., Knorr, U., Werhahn, K. J., Schlaug, G., Kunesch, E., Cohen, L. G., Seitz, R. J., & Benecke, R. (1998). Multimodal output mapping of human central motor representation on different spatial scales. The Journal of Physiology, 512(1), 163–179. 10.1111/j.1469-7793.1998.163bf.x 9729626 PMC2231178

[IMAG.a.1056-b12] Dannhauer, M., Huang, Z., Beynel, L., Wood, E., Bukhari-Parlakturk, N., & Peterchev, A. V. (2022). TAP: Targeting and analysis pipeline for optimization and verification of coil placement in transcranial magnetic stimulation. Journal of Neural Engineering, 19(2), 026050. 10.1088/1741-2552/ac63a4 PMC9131512 35377345

[IMAG.a.1056-b13] Diester, I., Bartos, M., Bödecker, J., Kortylewski, A., Leibold, C., Letzkus, J., Nour, M. M., Schönauer, M., Straw, A., Valada, A., Vlachos, A., & Brox, T. (2024). Internal world models in humans, animals, and AI. Neuron, 112(14), 2265–2268. 10.1016/j.neuron.2024.06.019 39024919

[IMAG.a.1056-b14] Eldar, Y., Lindenbaum, M., Porat, M., & Zeevi, Y. Y. (1997). The farthest point strategy for progressive image sampling. IEEE Transactions on Image Processing, 6(9), 1305–1315. 10.1109/83.623193 18283019

[IMAG.a.1056-b15] Fox, P. T., Narayana, S., Tandon, N., Sandoval, H., Fox, S. P., Kochunov, P., & Lancaster, J. L. (2004). Column-based model of electric field excitation of cerebral cortex. Human Brain Mapping, 22(1), 1–14. 10.1002/hbm.20006 15083522 PMC6872111

[IMAG.a.1056-b16] Gomez–Tames, J., Laakso, I., Murakami, T., Ugawa, Y., & Hirata, A. (2020). Tms activation site estimation using multiscale realistic head models. Journal of Neural Engineering, 17(3), 036004. 10.1088/1741-2552/ab8ccf 32330914

[IMAG.a.1056-b17] Haddad, A. F., Young, J. S., Berger, M. S., & Tarapore, P. E. (2021). Preoperative applications of navigated transcranial magnetic stimulation. Frontiers in Neurology, 11, 628903. 10.3389/fneur.2020.628903 33551983 PMC7862711

[IMAG.a.1056-b18] Hasan, N. I., Dannhauer, M., Wang, D., Deng, Z.-D., & Gomez, L. J. (2025). Real-time computation of brain e-field for enhanced transcranial magnetic stimulation neuronavigation and optimization. Imaging Neuroscience, 3, imag_a_00412. 10.1162/imag_a_00412 PMC12319877 40800850

[IMAG.a.1056-b19] Jeltema, H.-R., Ohlerth, A.-K., de Wit, A., Wagemakers, M., Rofes, A., Bastiaanse, R., & Drost, G. (2021). Comparing navigated transcranial magnetic stimulation mapping and “gold standard” direct cortical stimulation mapping in neurosurgery: A systematic review. Neurosurgical Review, 44, 1903–1920. 10.1007/s10143-020-01397-x 33009990 PMC8338816

[IMAG.a.1056-b20] Jing, Y., Numssen, O., Weise, K., Kalloch, B., Buchberger, L., Haueisen, J., Hartwigsen, G., & Knösche, T. R. (2023). Modeling the effects of transcranial magnetic stimulation on spatial attention. Physics in Medicine & Biology, 68(21), 214001. 10.1088/1361-6560/acff34 37783213

[IMAG.a.1056-b21] Julkunen, P. (2019). Mobile application for adaptive threshold hunting in transcranial magnetic stimulation. IEEE Transactions on Neural Systems and Rehabilitation Engineering, 27(8), 1504–1510. 10.1109/tnsre.2019.2925904 31265403

[IMAG.a.1056-b22] Kim, H., Kim, J., Lee, H.-J., Lee, J., Na, Y., Chang, W. H., & Kim, Y.-H. (2021). Optimal stimulation site for rTMS to improve motor function: Anatomical hand knob vs. hand motor hotspot. Neuroscience Letters, 740, 135424. 10.1016/j.neulet.2020.135424 33075419

[IMAG.a.1056-b23] Koponen, L. M., & Peterchev, A. V. (2022). Preventing misestimation of transcranial magnetic stimulation motor threshold with mtat 2.0. Brain Stimulation, 15(5), 1073–1076. 10.1016/j.brs.2022.07.057 35940558 PMC9869889

[IMAG.a.1056-b24] Krieg, T. D., Salinas, F. S., Narayana, S., Fox, P. T., & Mogul, D. J. (2015). Computational and experimental analysis of TMS-induced electric field vectors critical to neuronal activation. Journal of Neural Engineering, 12(4), 046014. 10.1088/1741-2560/12/4/046014 26052136

[IMAG.a.1056-b25] Krings, T., Buchbinder, B. R., Butler, W. E., Chiappa, K. H., Jiang, H. J., Rosen, B. R., & Cosgrove, G. R. (1997). Stereotactic transcranial magnetic stimulation: Correlation with direct electrical cortical stimulation. Neurosurgery, 41(6), 1319–1326. 10.1097/00006123-199712000-00016 9402583

[IMAG.a.1056-b26] Krings, T., Chiappa, K. H., Foltys, H., Reinges, M. H., Cosgrove, R. G., & Thron, A. (2001). Introducing navigated transcranial magnetic stimulation as a refined brain mapping methodology. Neurosurgical Review, 24, 171–179. 10.1007/s101430100151 11778822

[IMAG.a.1056-b27] Laakso, I., & Hirata, A. (2012). Fast multigrid-based computation of the induced electric field for transcranial magnetic stimulation. Physics in Medicine & Biology, 57(23), 7753. 10.1088/0031-9155/57/23/7753 23128377

[IMAG.a.1056-b28] Laakso, I., Murakami, T., Hirata, A., & Ugawa, Y. (2018). Where and what TMS activates: Experiments and modeling. Brain Stimulation, 11(1), 166–174. 10.1016/j.brs.2017.09.011 29030110

[IMAG.a.1056-b29] Mayka, M. A., Corcos, D. M., Leurgans, S. E., & Vaillancourt, D. E. (2006). Three-dimensional locations and boundaries of motor and premotor cortices as defined by functional brain imaging: A meta-analysis. NeuroImage, 31(4), 1453–1474. 10.1016/j.neuroimage.2006.02.004 16571375 PMC2034289

[IMAG.a.1056-b30] Mitchell, D. P. (1991). Spectrally optimal sampling for distribution ray tracing. Proceedings of the 18th Annual Conference on Computer Graphics and Interactive Techniques, 157–164. 10.1145/122718.122736

[IMAG.a.1056-b31] Moré, J. J. (2006). The levenberg-marquardt algorithm: Implementation and theory. Numerical Analysis: Proceedings of the Biennial Conference held at Dundee, June 28–July 1, 1977, 105–116. 10.1007/bfb0067700

[IMAG.a.1056-b32] Mueller, J. K., Grigsby, E. M., Prevosto, V., Petraglia III, F. W., Rao, H., Deng, Z.-D., Peterchev, A. V., Sommer, M. A., Egner, T., Platt, M. L., & Grill, W. M. (2014). Simultaneous transcranial magnetic stimulation and single-neuron recording in alert non-human primates. Nature Neuroscience, 17(8), 1130–1136. 10.1038/nn.3751 24974797 PMC4115015

[IMAG.a.1056-b33] Muir, M., Gadot, R., Prinsloo, S., Michener, H., Traylor, J., Athukuri, P., Tummala, S., Kumar, V. A., & Prabhu, S. S. (2022). Comparative study of preoperative functional imaging combined with tractography for prediction of iatrogenic motor deficits. Journal of Neurosurgery, 139(1), 65–72. 10.3171/2022.10.jns221684 36433877

[IMAG.a.1056-b34] Nazarova, M., Novikov, P., Ivanina, E., Kozlova, K., Dobrynina, L., & Nikulin, V. V. (2021). Mapping of multiple muscles with transcranial magnetic stimulation: Absolute and relative test–retest reliability. Human Brain Mapping, 42(8), 2508–2528. 10.1002/hbm.25383 33682975 PMC8090785

[IMAG.a.1056-b35] Numssen, O., Zier, A.-L., Thielscher, A., Hartwigsen, G., Knösche, T. R., & Weise, K. (2021). Efficient high-resolution TMS mapping of the human motor cortex by nonlinear regression. NeuroImage, 245, 118654. 10.1016/j.neuroimage.2021.118654 34653612

[IMAG.a.1056-b36] Oldfield, R. C. (1971). The assessment and analysis of handedness: The Edinburgh inventory. Neuropsychologia, 9(1), 97–113. 10.1016/0028-3932(71)90067-4 5146491

[IMAG.a.1056-b37] Opitz, A., Fox, M. D., Craddock, R. C., Colcombe, S., & Milham, M. P. (2016). An integrated framework for targeting functional networks via transcranial magnetic stimulation. NeuroImage, 127, 86–96. 10.1016/j.neuroimage.2015.11.040 26608241 PMC4836057

[IMAG.a.1056-b38] Opitz, A., Windhoff, M., Heidemann, R. M., Turner, R., & Thielscher, A. (2011). How the brain tissue shapes the electric field induced by transcranial magnetic stimulation. NeuroImage, 58(3), 849–859. 10.1016/j.neuroimage.2011.06.069 21749927

[IMAG.a.1056-b39] Pentland, A. (1980). Maximum-likelihood estimation-the best pest. 10.3758/bf03204398 7465322

[IMAG.a.1056-b40] Picht, T., Schmidt, S., Brandt, S., Frey, D., Hannula, H., Neuvonen, T., Karhu, J., Vajkoczy, P., & Suess, O. (2011). Preoperative functional mapping for rolandic brain tumor surgery: Comparison of navigated transcranial magnetic stimulation to direct cortical stimulation. Neurosurgery, 69(3), 581–589. 10.1227/neu.0b013e3182181b89 21430587

[IMAG.a.1056-b41] Raffa, G., Scibilia, A., Conti, A., Ricciardo, G., Rizzo, V., Morelli, A., Angileri, F. F., Cardali, S. M., & Germano, A. (2019). The role of navigated transcranial magnetic stimulation for surgery of motor-eloquent brain tumors: A systematic review and meta-analysis. Clinical Neurology and Neurosurgery, 180, 7–17. 10.1016/j.clineuro.2019.03.003 30870762

[IMAG.a.1056-b42] Raffin, E., Pellegrino, G., Di Lazzaro, V., Thielscher, A., & Siebner, H. R. (2015). Bringing transcranial mapping into shape: Sulcus-aligned mapping captures motor somatotopy in human primary motor hand area. NeuroImage, 120, 164–175. 10.1016/j.neuroimage.2015.07.024 26188259

[IMAG.a.1056-b43] Reijonen, J., Säisänen, L., Pitkänen, M., Julkunen, M., Ilmoniemi, R. J., Nieminen, P., & Julkunen, P. (2022). Minimum-norm estimation of TMS-activated motor cortical sites in realistic head and brain geometry. IEEE Transactions on Neural Systems and Rehabilitation Engineering, 30, 441–454. 10.1109/tnsre.2022.3151678 35167479

[IMAG.a.1056-b44] Romero, M. C., Davare, M., Armendariz, M., & Janssen, P. (2019). Neural effects of transcranial magnetic stimulation at the single-cell level. Nature Communications, 10(1), 2642. 10.1038/s41467-019-10638-7 PMC6572776 31201331

[IMAG.a.1056-b45] Rossini, P. M., Burke, D., Chen, R., Cohen, L. G., Daskalakis, Z., Di Iorio, R., Di Lazzaro, V., Ferreri, F., Fitzgerald, P., George, M. S., Hallett, M., Lefaucheur, J. P., Langguth, B., Matsumoto, H., Miniussi, C., Nitsche, M. A., Pascual-Leone, A., Paulus, W., Rossi, S., … Ziemann, U. (2015). Non-invasive electrical and magnetic stimulation of the brain, spinal cord, roots and peripheral nerves: Basic principles and procedures for routine clinical and research application. An updated report from an IFCN committee. Clinical Neurophysiology, 126(6), 1071–1107. 10.1016/j.clinph.2015.02.001 25797650 PMC6350257

[IMAG.a.1056-b46] Rothwell, J. C., Thompson, P. D., Day, B. L., Dick, J., Kachi, T., Cowan, J., & Marsden, C. D. (1987). Motor cortex stimulation in intact man: 1. general characteristics of EMG responses in different muscles. Brain, 110(5), 1173–1190. 10.1093/brain/110.5.1173 3676697

[IMAG.a.1056-b47] Saatlou, F. H., Rogasch, N. C., McNair, N. A., Biabani, M., Pillen, S. D., Marshall, T. R., & Bergmann, T. O. (2018). Magic: An open-source MATLAB toolbox for external control of transcranial magnetic stimulation devices. Brain Stimulation, 11(5), 1189–1191. 10.1016/j.brs.2018.05.015 29885859

[IMAG.a.1056-b48] Sartori, L., Meneghini, G., Caliri, S., De Nardi, G., Facchini, S., Baro, V., Ferreri, F., Corbetta, M., D’Avella, D., & Landi, A. (2023). Pre-surgical mapping of motor and language functions in brain tumor patients using navigated transcranial magnetic stimulation. Brain Stimulation, 16(1), 261. 10.1016/j.brs.2023.01.431

[IMAG.a.1056-b49] Siebner, H. R., Funke, K., Aberra, A. S., Antal, A., Bestmann, S., Chen, R., Classen, J., Davare, M., Di Lazzaro, V., Fox, P. T., Hallett, M., Karabanov, A. N., Kesselheim, J., Beck, M. M., Koch, G., Liebetanz, D., Meunier, S., Miniussi, C., Paulus, W., … Ugawa, Y. (2022). Transcranial magnetic stimulation of the brain: What is stimulated?—A consensus and critical position paper. Clinical Neurophysiology, 140, 59–97. 10.1016/j.clinph.2022.04.022 35738037 PMC9753778

[IMAG.a.1056-b50] Sollmann, N., Goblirsch-Kolb, M. F., Ille, S., Butenschoen, V. M., Boeckh-Behrens, T., Meyer, B., Ringel, F., & Krieg, S. M. (2016). Comparison between electric-field-navigated and line-navigated TMS for cortical motor mapping in patients with brain tumors. Acta Neurochirurgica, 158, 2277–2289. 10.1007/s00701-016-2970-6 27722947

[IMAG.a.1056-b51] Thielscher, A., Antunes, A., & Saturnino, G. B. (2015). Field modeling for transcranial magnetic stimulation: A useful tool to understand the physiological effects of TMS? 2015 37th Annual International Conference of the IEEE Engineering in Medicine and Biology Society (EMBC), 222–225. 10.1109/embc.2015.7318340 26736240

[IMAG.a.1056-b52] Turi, Z., Hananeia, N., Shirinpour, S., Opitz, A., Jedlicka, P., & Vlachos, A. (2022). Dosing transcranial magnetic stimulation of the primary motor and dorsolateral prefrontal cortices with multi-scale modeling. Frontiers in Neuroscience, 16, 929814. 10.3389/fnins.2022.929814 35898411 PMC9309210

[IMAG.a.1056-b53] Umana, G. E., Scalia, G., Graziano, F., Maugeri, R., Alberio, N., Barone, F., Crea, A., Fagone, S., Giammalva, G. R., Brunasso, L., Costanzo, R., Paolini, F., Gerardi, R. M., Tumbiolo, S., Cicero, S., Federico Nicoletti, G., & Iacopino, D. G. (2021). Navigated transcranial magnetic stimulation motor mapping usefulness in the surgical management of patients affected by brain tumors in eloquent areas: A systematic review and meta-analysis. Frontiers in Neurology, 12, 644198. 10.3389/fneur.2021.644198 33746895 PMC7970041

[IMAG.a.1056-b54] Wang, D., Hasan, N. I., Dannhauer, M., Yucel, A. C., & Gomez, L. J. (2023). Fast computational e-field dosimetry for transcranial magnetic stimulation using adaptive cross approximation and auxiliary dipole method (ACA-ADM). NeuroImage, 267, 119850. 10.1016/j.neuroimage.2022.119850 36603745 PMC11658687

[IMAG.a.1056-b55] Wassermann, E. M., McShane, L. M., Hallett, M., & Cohen, L. G. (1992). Noninvasive mapping of muscle representations in human motor cortex. Electroencephalography and Clinical Neurophysiology/Evoked Potentials Section, 85(1), 1–8. 10.1016/0168-5597(92)90094-r 1371738

[IMAG.a.1056-b56] Weise, K., Numssen, O., Kalloch, B., Zier, A. L., Thielscher, A., Haueisen, J., Hartwigsen, G., & Knösche, T. R. (2023). Precise motor mapping with transcranial magnetic stimulation. Nature Protocols, 18(2), 293–318. 10.1038/s41596-022-00776-6 36460808

[IMAG.a.1056-b57] Weise, K., Numssen, O., Thielscher, A., Hartwigsen, G., & Knösche, T. R. (2020). A novel approach to localize cortical TMS effects. NeuroImage, 209, 116486. 10.1016/j.neuroimage.2019.116486 31877374

