# Supplementary material for: Efficient prospective electric field-informed localization of motor cortical targets of transcranial magnetic stimulation
Source: Imaging Neurosci (Camb). 2025 Dec 19;3:IMAG.a.1056. doi: 10.1162/IMAG.a.1056 (PMC12720167; doi:10.1162/IMAG.a.1056)
Supplement: Supplementary Material [file IMAG.a.1056_supp.pdf]

Supplemental Information to Efficient Prospective Electric Field-Informed Localization of Motor Cortical Targets of Transcranial Magnetic Stimulation

David Luis Schultheiss[1,10, *], Zsolt Turi[2, *], Srilekha Marmavula[2], Peter Christoph Reinacher[3,4], Theo Demerath[5], Jakob Straehle[6], Joschka Boedecker[1,10], Matthias Mittner[7,8, **], and Andreas Vlachos[2,9,10, **]

[1]Neurobotics Lab, Department of Computer Science, University of Freiburg, Freiburg, Germany.

[2]Department of Neuroanatomy, Institute of Anatomy and Cell Biology, Faculty of Medicine, University of Freiburg, Freiburg, Germany.

[3]Department of Stereotactic and Functional Neurosurgery, Medical Center - University of Freiburg, Faculty of Medicine, University of Freiburg, Freiburg, Germany.

[4]Fraunhofer Institute for Laser Technology (ILT), Aachen, Germany.

[5]Department of Neuroradiology, Medical Center-University of Freiburg, Faculty of Medicine, University of Freiburg, Freiburg, Germany.

[6]Department of Neurosurgery, Medical Center-University of Freiburg, Faculty of Medicine, University of Freiburg, Freiburg, Germany.

[7]Institute for Psychology, Norwegian University of Science and Technology, 7491 Trondheim, Norway.

[8]Institute for Psychology, UiT-The Arctic University of Norway, 9019 Tromsø, Norway.

[9]Center for Basics in NeuroModulation (NeuroModulBasics), Faculty of Medicine, University of Freiburg, Freiburg, Germany.

[10]Center BrainLinks-BrainTools, University of Freiburg, Freiburg, Germany.

andreas.vlachos@anat.uni-freiburg.de.

[*]Co-first authorship

[**]Co-last authorship

2025

Appendix A Simulation resolution

A.1 Angular symmetry

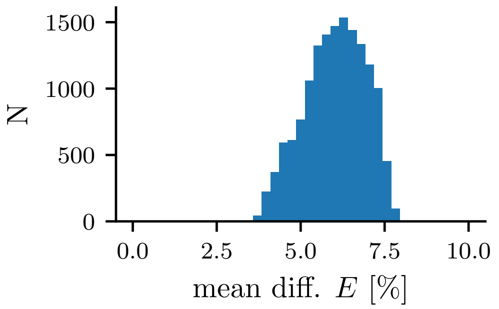

Figure A.1: **Angular symmetry.** Histogram of mean relative difference over 14911 compartments. Differences of 31320 E-fields and their 180° rotated versions are averaged per compartment.

In this study we very using a figure-of-eight coil. Thus, the left and right side of the coil are symmetric and a rotation of the coil by 180° should result in a similar induced E-field. With this geometric property, the simulations needed to generate a dataset per participant can be cut to half. Differences between two symmetric positions only arise because of asymmetries in power lines and coil cases. To test whether this is reflected in the data, we calculated the mean relative error of the E-field maps and their 180° rotated counterparts for each compartment of the ROI. Figure A.1 shows that for all compartments the errors is <10%. Thus, we only took into account rotation angles from 0° to 179° for this study.

A.2 Resolution for FPS

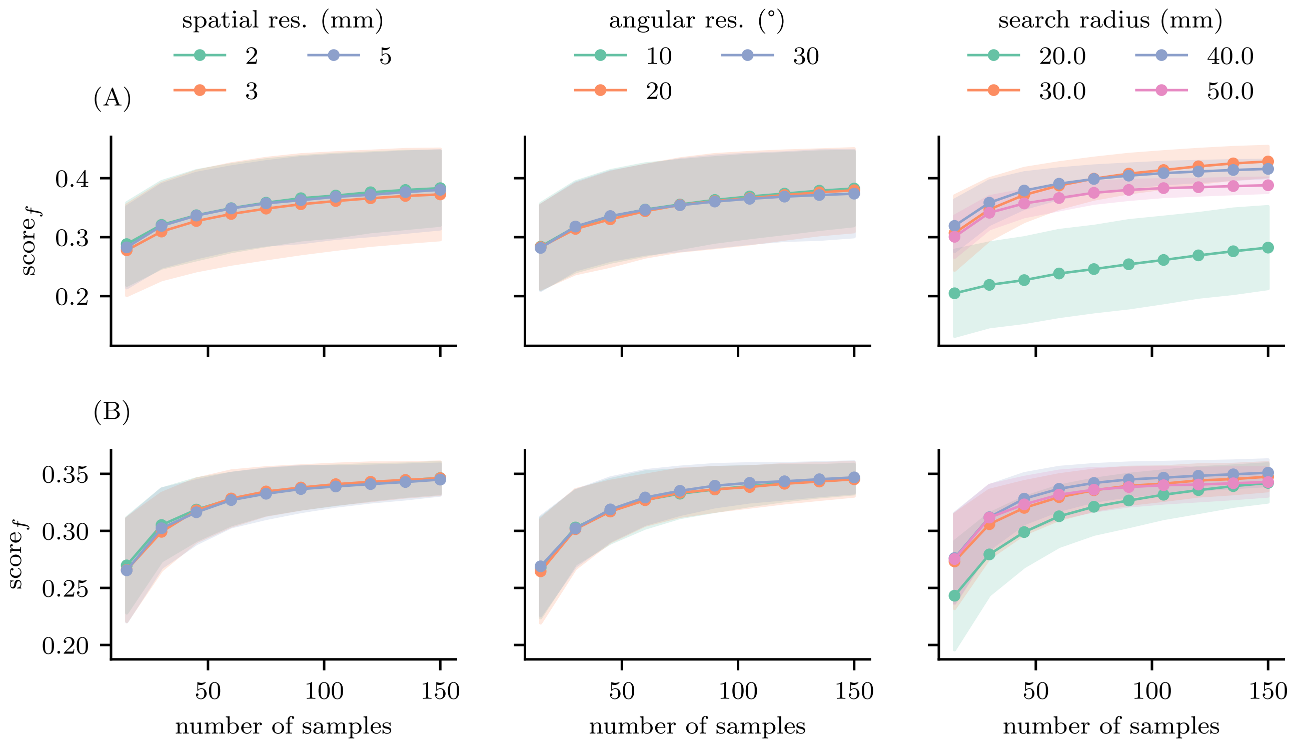

Figure A.2: **FPS resolution.** We varied the spatial resolution, angular resolution, and search radius of the circular grid for generating the candidate E-field dataset for FPS. For each combination of resolution parameters, we tested how well the MGM of the synthetic MEP model could be recovered. We placed the hypothetical muscle representation at (A) sulcal crown in the center of the ROI and (B) a location at the edge of the ROI. Only the search radius has a significant effect on the quality of fit.

The size of the candidate dataset $\boldsymbol{C}$ depends heavily on the coil search radius, spatial resolution, and angular resolution of the grid used to generate all possible coil configurations. For example, a search radius of 50mm, angular resolution of 1° between 0 and 179°, and spatial resolution of 1mm results in a size of $N_c \approx 2.800.000$ samples. Because E-field simulations are computationally expensive, we examined which resolution is sufficient to have optimal performance of FPS on simulated data.

Figure A.2 shows that the goodness of fit is constant for spatial resolutions of 2mm, 3m, 5mm and also for angular resolutions of 10°, 20°, 30°. However, there are differences for various search radii with 30mm yielding the best results. For the spatial and angular resolutions, 5mm and 20° were adequate choices. Furthermore, we only considered angles of $a_c \in [0°, 180°)$ because of the almost symmetric geometry of the coil. This results in a much smaller candidate dataset of $N_c = 1017$ samples per participant which can be performed in a few hours on a standard computer.

Appendix B Synthetic MEP model parameter sweep

The default MEP model parameters were chosen to simulate realistic experimental conditions and to facilitate optimization. The saturation amplitude y_{max} was fixed at 1. The sigmoid's turning point on the abscissa x_0 is set to the median of all stimulation strengths $\boldsymbol{x}$ in the dataset. The slope of the sigmoid k was derived based on a reference value k_{ref} which corresponds to the steepness required for the sigmoid curve to reach 95% of y_{max} at the maximum value of stimulation strength x:

$$0.95 \cdot y_{max} = \frac{y_{max}}{1 + e^{-k_{ref}(\max \boldsymbol{x} - x_0)}} \tag{1}$$

$$k_{ref} \approx \frac{2.94}{\max \boldsymbol{x} - x_0} \tag{2}$$

To simulate a realistically steep response curve, the slope was set to $k = 2k_{ref}$. The MGM standard deviation σ (spread) was set to 3 mm, and the noise amplitude P was set to 0.3.

We repeated the experiments conducted in *Section 3.1* to study the effect of different MEP model parameters. Figure A.3 shows that FPS is outperforming random sampling of the noise amplitude $P = \{0.2, 0.3, 0.75\}$. We also observe, that performance only slowly decreases for larger noise amplitudes, making FPS very robust against MEP amplitude noise.

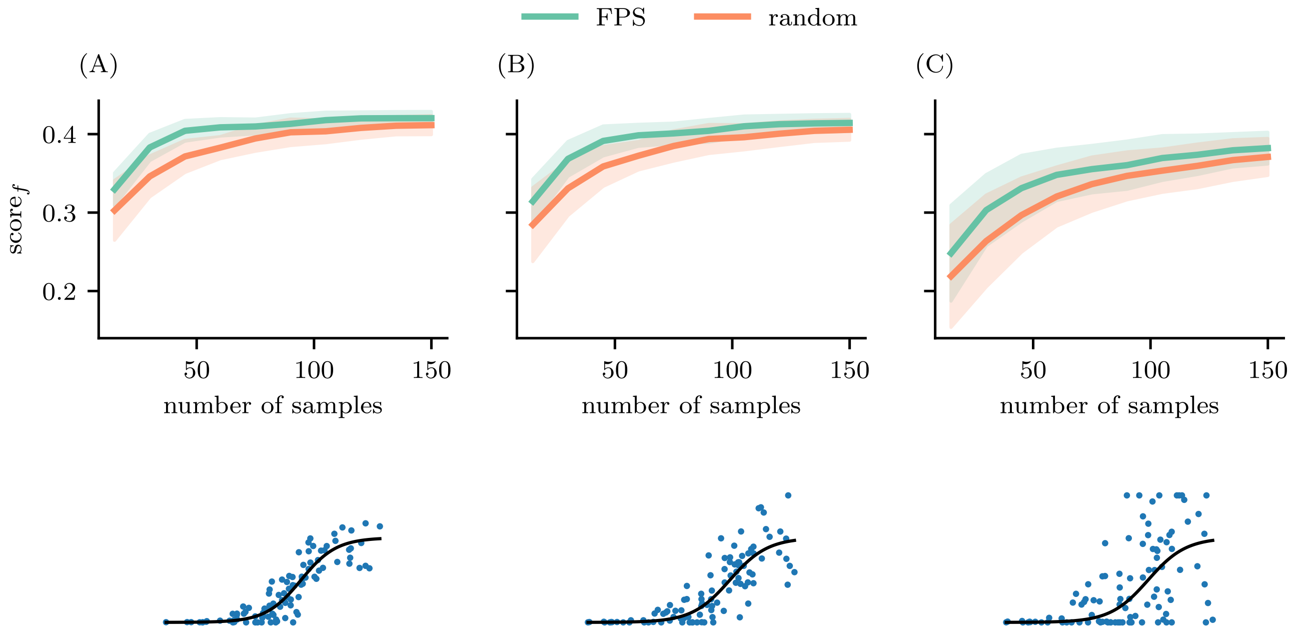

Figure A.3: **Effect of noise amplitude.** We replicated the synthetic data experiment for (A) small ($P = 0.2$), (B) medium ($P = 0.3$), and (C) large ($P = 0.75$) noise amplitudes. FPS constantly outperforms random sampling. For larger noise amplitudes the performance of both methods gets worse.

Figure A.4 illustrates the effect of muscle representation extension, represented by the MGM standard deviation σ. Again, FPS outperforms random sampling for all tested values $\sigma = \{1\,\text{mm}, 3\,\text{mm}, 6\,\text{mm}\}$. However, the scores indicate that performance drops rapidly with more focalized muscle representations. This hints at the limitation of combining R^2 values into a map as locations close to the true muscle representations are often co-stimulated and therefore also exhibit large R^2-values.

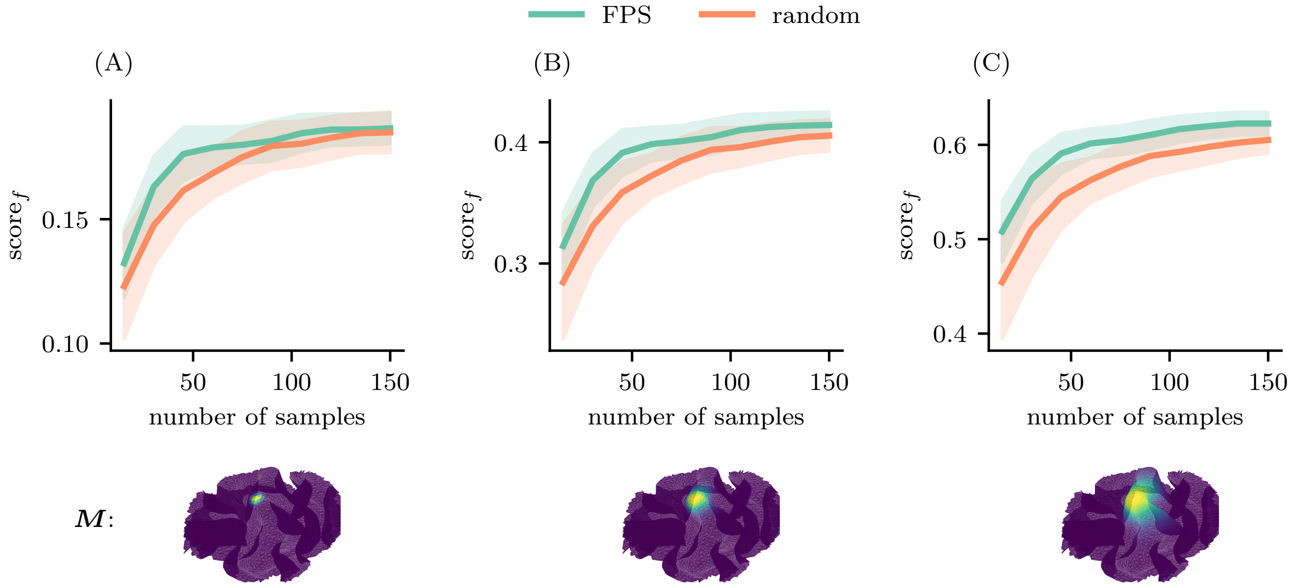

Figure A.4: **Effect of motor map spread.** We replicated the synthetic data experiment for (A) small ($\sigma = 1$ mm), (B) medium ($\sigma = 3$ mm), and (C) large ($\sigma = 6$ mm) muscle representation extension σ. FPS constantly outperforms random sampling. For smaller extensions the performance of both methods gets worse.

Lastly, we tested the effect of the sigmoid slope $k = \{k_{ref}, 2k_{ref}, 3k_{ref}\}$ on the performance as shown in Figure A.5. Again, FPS consistently outperforms random sampling. In general, k only has negligible effects on

the experiment.

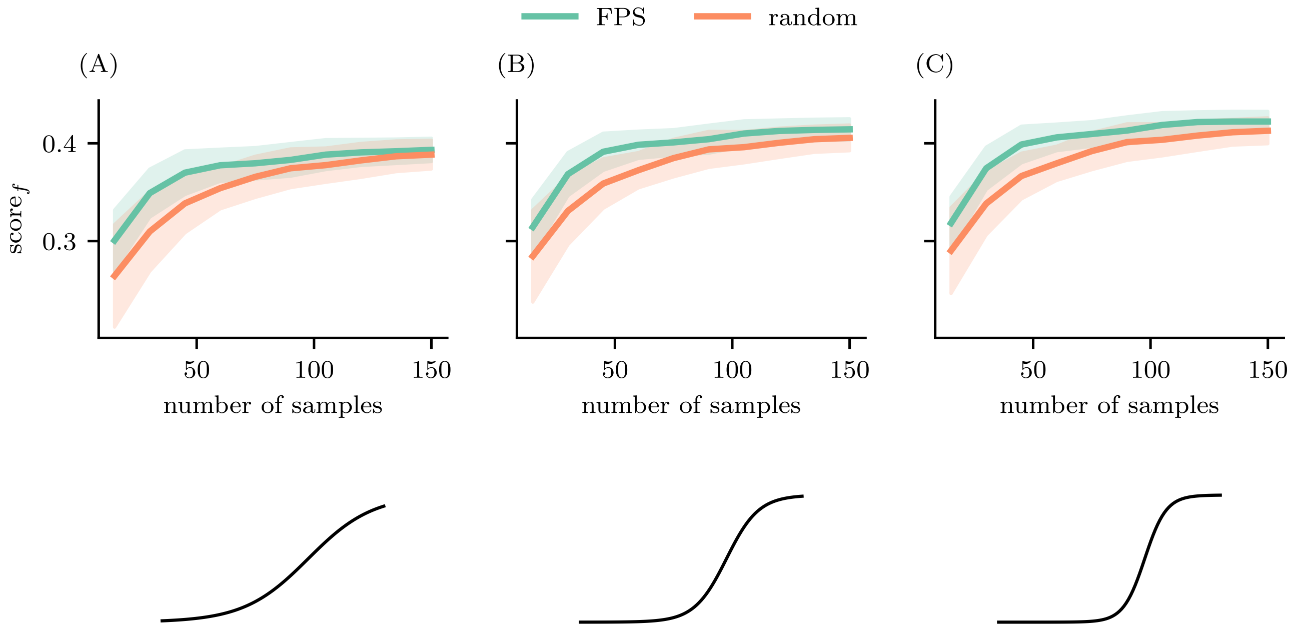

Figure A.5: **Effect of sigmoid slope.** We replicated the synthetic data experiment for (A) small ($k = k_{ref}$), (B) medium ($k = 2k_{ref}$), and (C) large ($k = 3k_{ref}$) sigmoid steepness k. FPS constantly outperforms random sampling. k only has neglectable effects on the experiment.

Appendix C Convergence to optimum in synthetic experiment

Figure A.6 shows that for all three simulated muscle representations, FPS converges faster to the upper limit than random sampling. Notably, both sampling methods converge to the optimal $\text{score}_f(\boldsymbol{R}^{\boldsymbol{2}}_{HR}, \hat{\boldsymbol{R}}^{\boldsymbol{2}})$ within the tested number of collected samples.

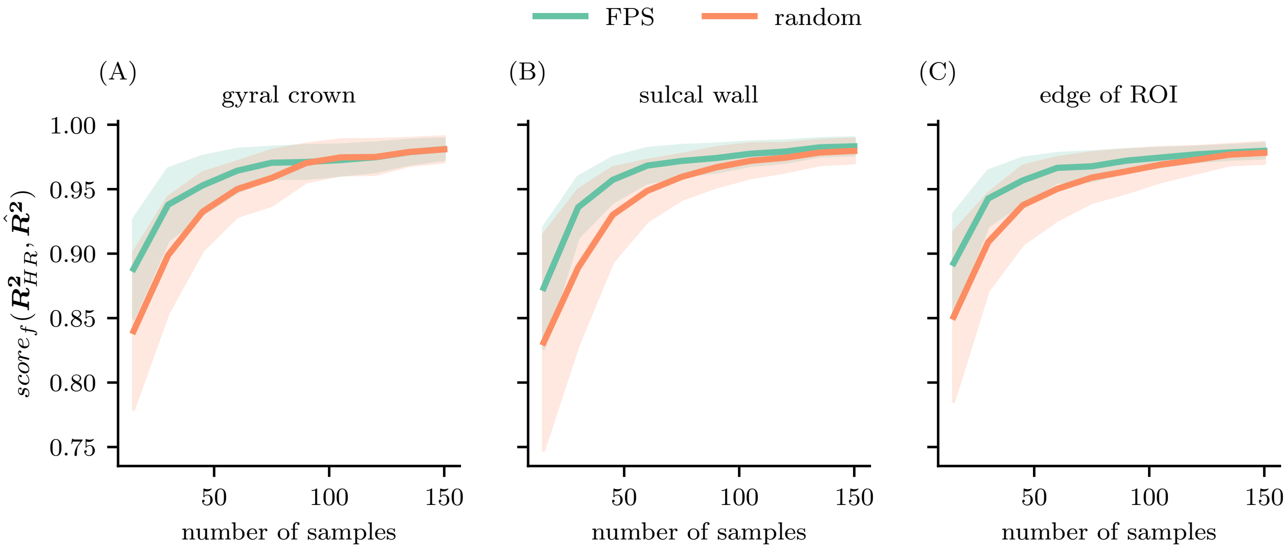

Figure A.6: **Subsampling from the synthetic data demonstrates faster convergence to the optimal fit with the FPS algorithm compared to the random sampling across all motor map locations.** We subsampled from a large dataset of simulated E-fields. The motor maps $\boldsymbol{M}$ used to generate noisy MEPs were placed at the gyral crown in the center of the ROI (A), the sulcal wall in the center of the ROI (B), and the gyral crown at the ROI edge (C). We fitted a high resolution (HR) map $\boldsymbol{R}^2_{HR}$ as an upper performance limit and tested how well $\boldsymbol{R}^2_{HR}$ could be recovered by computing $\text{score}_f(\boldsymbol{R}^2_{HR}, \hat{\boldsymbol{R}}^2)$ with increasing number of samples. The lines represent the mean across 100 runs, the shaded areas the standard deviation from the mean. FPS converges faster to the optimal fitting score than random sampling for all three simulated muscle representations.

Appendix D Motor mapping on previously published data

In their study Nummsen et al. (2021) conducted 1005 random stimulations and recorded the MEPs of first dorsal interosseous (FDI), the musculus abductor digiti minimi (ADM), and the musculus abductor pollicis brevis (APB). The data for one participant is publicly available. Thus, there exists a R^2-map for all three muscles retrieved from all 1005 stimulations. We call this the ground truth. We then subsampled the data randomly and with FPS and tested how similar the R^2-maps are for different numbers of subsamples.

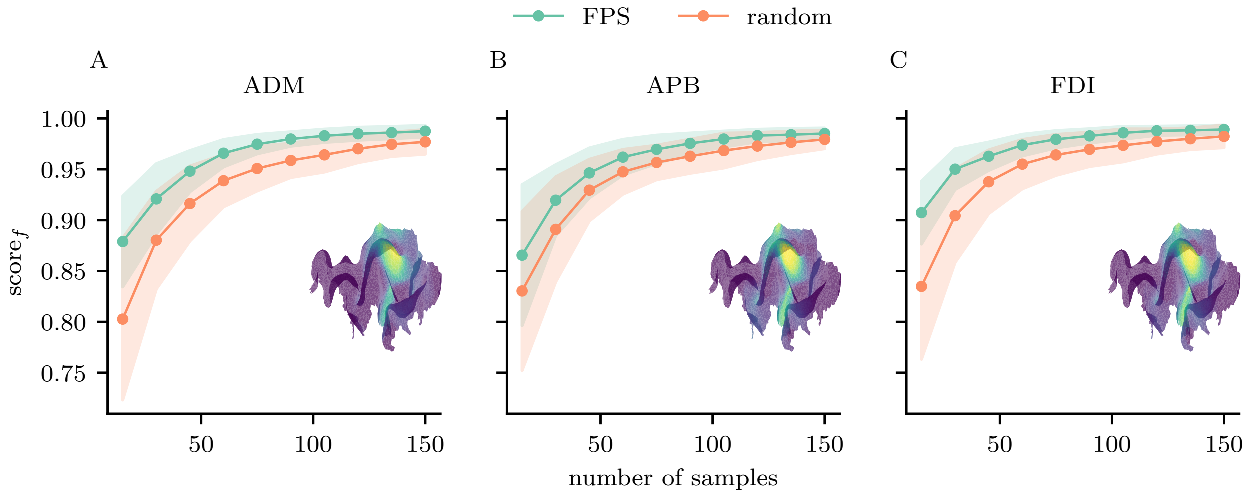

Figure A.7: **Subsampling larger dataset.** We subsampled a larger dataset from Numssen et al. (2021) containing MEP Measurements of three muscles for 1005 random coil configurations. For each muscle we measured the fitting score score$_f$ after every 20 added samples. As ground truth served motor maps fitted with all 1005 samples depicted as insets in the graph. The dots represent the mean across 100 random initializations and the shaded area the standard deviation form the mean. FPS outperforms random sampling for all three muscles, especially in the early sampling phase and has consistently lower variation across initialization

Figure A.7 shows that pre-selecting E-fields based on FPS yields better results for all three muscles. To reach an overlap of 95%, only 50 FPS samples are needed for all three muscles. With random sampling up to 100 stimulations are needed to reach the same score. This performance gap is closing as the number of samples increases. However, the variance across random initialization (100) of the starting sample leads to larger variance in random sampling than in FPS, making FPS the more robust method.

Appendix E MNI coordinate statistics

**MNI Coordinate Statistics**		
**Condition**	**Mean (x, y, z)**	**Std (x, y, z)**
All	[−36.18, −13.64, 57.80]	[4.57, 7.50, 4.85]
Random	[−38.48, −14.13, 61.36]	[3.74, 8.28, 6.28]
FPS	[−37.44, −14.00, 59.15]	[5.06, 7.20, 5.24]

Table A.1: Mean and standard deviation of MNI coordinates (x, y, z) for each condition.

References

Numssen, O., Zier, A.-L., Thielscher, A., Hartwigsen, G., Knösche, T. R., & Weise, K. (2021). Efficient high-resolution tms mapping of the human motor cortex by nonlinear regression. *NeuroImage*, *245*, 118654.
